# Multi-omics study of silicosis reveals the potential therapeutic targets PGD_2_ and TXA_2_

**DOI:** 10.7150/thno.47627

**Published:** 2021-01-01

**Authors:** Junling Pang, Xianmei Qi, Ya Luo, Xiaona Li, Ting Shu, Baicun Li, Meiyue Song, Ying Liu, Dong Wei, Jingyu Chen, Jing Wang, Chen Wang

**Affiliations:** 1 State Key Laboratory of Medical Molecular Biology, Institute of Basic Medical Sciences, Chinese Academy of Medical Sciences, Department of Pathophysiology, Peking Union Medical College, Beijing 100730, China; 2 State Key Laboratory of Medical Molecular Biology, Institute of Basic Medical Sciences, Chinese Academy of Medical Sciences, Department of Physiology, Peking Union Medical College, Beijing 100730, China; 3 Wuxi Lung Transplantation Center, Wuxi People's Hospital Affiliated with Nanjing Medical University, Wuxi 214023, China; 4 Department of Thoracic Surgery and Lung Transplantation, China-Japan Friendship Hospital, Beijing 100029, China; 5 Beijing University of Chinese Medicine, Beijing 100029, China

**Keywords:** silicosis, multi-omics, PGD_2_, TXA_2_, Ramatroban

## Abstract

**Rationale**: Silicosis is a severe occupational lung disease. Current treatments for silicosis have highly limited availability (*i.e.,* lung transplantation) or, do not effectively prolong patient survival time (*i.e.,* lung lavage). There is thus an urgent clinical need for effective drugs to retard the progression of silicosis.

**Methods**: To systematically characterize the molecular changes associated with silicosis and to discover potential therapeutic targets, we conducted a transcriptomics analysis of human lung tissues acquired during transplantation, which was integrated with transcriptomics and metabolomics analyses of silicosis mouse lungs. The results from the multi-omics analyses were then verified by qPCR, western blot, and immunohistochemistry. The effect of Ramatroban on the progression of silicosis was evaluated in a silica-induced mouse model.

**Results**: Wide metabolic alterations were found in lungs from both human patients and mice with silicosis. Targeted metabolite quantification and validation of expression of their synthases revealed that arachidonic acid (AA) pathway metabolites, prostaglandin D_2_ (PGD_2_) and thromboxane A_2_ (TXA_2_), were significantly up-regulated in silicosis lungs. We further examined the effect of Ramatroban, a clinical antagonist of both PGD_2_ and TXA_2_ receptors, on treating silicosis using a mouse model. The results showed that Ramatroban significantly alleviated silica-induced pulmonary inflammation, fibrosis, and cardiopulmonary dysfunction compared with the control group.

**Conclusion**: Our results revealed the importance of AA metabolic reprogramming, especially PGD_2_ and TXA_2_ in the progression of silicosis. By blocking the receptors of these two prostanoids, Ramatroban may be a novel potential therapeutic drug to inhibit the progression of silicosis.

## Introduction

Silicosis is a potentially fatal lung disease caused by occupational exposure to respirable crystalline silica. As one of the leading occupational diseases worldwide, silicosis is currently incurable, which seriously threatens public health 1. Due to emerging modern industries, such as sandblasting denim jeans, stone fabrication and jewellery polishing, the incidence of silicosis is increasing both in developing and some developed countries 2, 3. Currently, the clinical treatments for this disease are limited. Lung lavage can remove sputum, secretions, dust, and fibrocytokines from the airway, which may improve symptoms but does not reverse pulmonary fibrosis or prolong survival time. Moreover, it typically works best in the early stage of this disease, when most of the inhaled dust still stays in the pulmonary alveoli 4. Although lung transplantation may potentially provide the longest survival time, it cannot be commonly used due to the low availability of lung donors and high operation cost. Therefore, it is urgent to identify effective drugs to alleviate the progression of silicosis and reduce the mortality of late-stage silicosis.

The progression of silicosis largely entails both a chronic inflammatory process and an aggravation of fibrotic process 1, 5. Various cells and factors have been reported to participate in the crystalline silica-triggered pathogenesis of silicosis 6. Growing studies reveal that autophagy-lysosomal system dysfunction in alveolar macrophages (AMs) involves in silica-induced inflammatory and fibrotic process 7-9. Moreover, the recruitment of lymphocytes and neutrophils are reported to provoke an inflammation response that afterwards leads to fibrosis 10-13. Multiple factors were found to participate in the inflammatory or fibrotic reactions 14, such as interleukin-6 (IL-6), interleukin-1 beta (IL-1β), tumor necrosis factor-alpha (TNF-α), transforming growth factor-beta (TGF-β), and insulin-like growth factor-1 (IGF-1). In spite of the progress toward a mechanistic understanding of silicosis 15-17, no effective drugs have yet been approved to retard the disease progression from chronic inflammation to lung fibrosis.

Recently, multi-omics approaches are increasingly popular for exploring underlying pathogenesis of diseases and uncovering critical therapeutic targets. Compared with traditional hypothesis-driven methods, multi-omics approaches are more comprehensive means to unearth molecular alterations and facilitate deeper insights into disease pathogenesis 18. Here, we conducted transcriptomics using the lung tissues from human patients, combining with transcriptomics and metabolomic analyses in mouse silicosis lungs to reveal potential therapeutic targets in silicosis progression. The reprogramming of arachidonic acid (AA) metabolic pathway was found remarkable in the wide changes of gene expression and metabolic programming throughout silicosis progression. Furthermore, we confirmed the crucial roles of the AA pathway metabolites prostaglandin D_2_ (PGD_2_) and thromboxane A_2_ (TXA_2_) in silicosis development, and demonstrated the therapeutic effects of Ramatroban in alleviating silica-induced pulmonary inflammation and fibrosis as well as cardiopulmonary dysfunction.

## Materials and Methods

A detailed description of the materials and methods is provided in the online supplementary materials.

### Human specimens

Silicosis lung tissues were collected from advanced silicosis patients who received lung transplantation, of which 90% patients are male. Subject characteristics were summarized in Table S1. Control lung tissues were the resected parts of the lungs from healthy donors removed due to size incompatibility during lung transplantation.

### Animal model

Silicosis model was constructed by a single intratracheal instillation of 8 mg of silica in 40 μL sterile phosphate-buffered saline (PBS) in male C57BL/6J mice of 8-10 weeks' old. The control group mice received an equal volume of sterile PBS. Six mice from each group were killed at different time points (3, 6, and 9 weeks) to assess the body weight, pulmonary function, and the status of lung inflammation and fibrosis. The same lung tissues were concurrently used for RNA isolation and RNA sequencing (RNA-seq).

To evaluate the effect of Ramatroban, mice were randomly divided into four groups (n = 8-10 mice per group): (a) PBS + vehicle, (b) PBS + Ramatroban, (c) Silica + vehicle, and (d) Silica + Ramatroban. Ramatroban or vehicle was given every day via tail vein injection starting after 3 weeks of silica / PBS instillation. Cardiopulmonary function was assessed, and mouse lungs were harvested to detect the status of lung inflammation and fibrosis at 3 weeks post-treatment.

### RNA-seq and bioinformatic analysis

The integrity and purity of RNA was checked prior to library preparations. A NEBNext® Ultra^TM^ RNA Library Prep Kit for Illumina was used according to the manufacturer's recommendations to create the sequencing libraries, which were then sequenced in 150-bp paired-end reads using an Illumina Hiseq platform (Novogene Co., Ltd, Beijing, China).

The cleaned reads of each sample were aligned to their respective reference genomes (human: GRCh38; mouse: GRCm38) using HISAT2 software with the default parameters 19. The read counts of each gene were then calculated using GenomicFeatures and GenomicAlignments packages in R 20. The gene expression levels (Fragments per Kilobase Million; FPKM) and the significance tests for differentially expressed genes were obtained through DESeq2 package 21. The Short Time-series Expression Miner (STEM) was used to compare and visualize gene expression profiles across the time points 22. The Gene Ontology (GO) and Kyoto Encyclopedia of Genes and Genomes (KEGG) pathway enrichment was conducted using the R package *clusterProfiler* 23. R packages, *GOplot* and* enrichplot*, were used for visualization of the enrichment results 24, 25.

### Untargeted/Targeted metabolomics analysis

Sample preparation and metabolic profiling were performed with standard procedures in cooperation with Core Facility of Instruments of Institute of Basic Medical Sciences, CAMS (Beijing, China), and Shanghai BIOTREE Biological Technology Co., Ltd. (Shanghai, China).

### Statistical analysis

Data sets were evaluated for normal distribution using D'Agostino-Pearson test, and presented as mean ± standard error of the mean (SEM). Statistical significance was determined by either a two-tailed unpaired Student's *t*-test (for 2 groups), or 2-way ANOVA (for grouped comparisons). In datasets operated by ANOVA, the Bonferroni post-hoc test was adopted. A p-value less than 0.05 was considered statistically significant. All statistical analysis was performed with Prism version 8.0 (GraphPad, La Jolla, CA, USA).

## Results

### RNA-seq analysis reveals metabolic reprogramming in the lungs of silicosis patients

**Figure 1:**
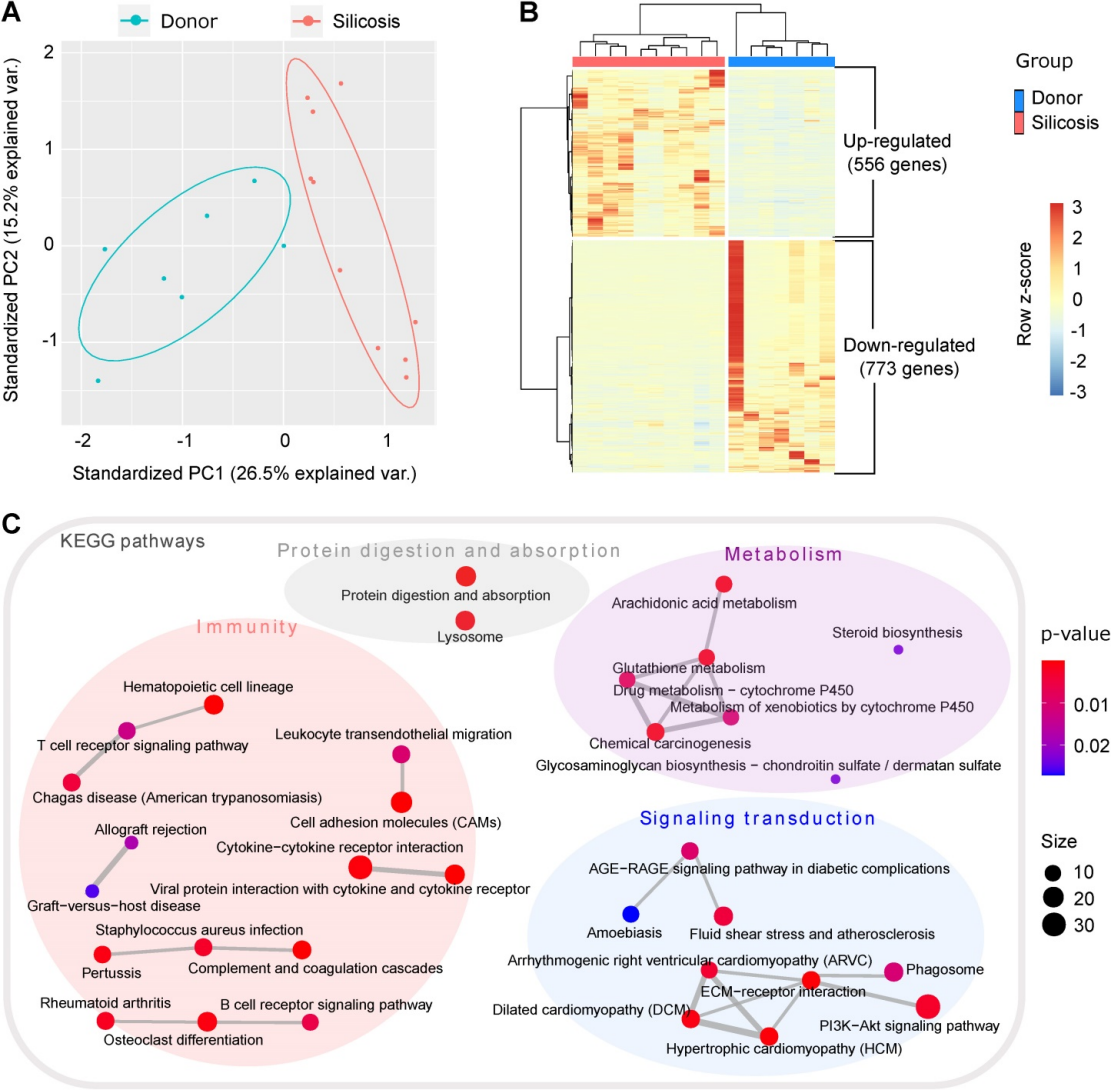
Transcriptomics analysis of lungs from silicosis patients and healthy donors. (A) Principal component analysis of the samples. The red dots indicate lung samples from silicosis patients (n = 10) and the blue dots represent lung samples from healthy donors (n = 7). (B) Heatmap showing the differentially expressed genes between silicosis and healthy lungs. Each column represents a sample and each row a gene. The expression of genes was scaled using z-score. (C) Significantly enriched KEGG items of the differential genes. Four super classes were highlighted based on the nature of the pathways. KEGG, kyoto encyclopedia of genes and genomes.

In order to systemically understand the molecular changes of silicosis, we performed RNA-seq for human lung tissues from ten advanced silicosis patients and seven healthy donors. Through principal component analysis (PCA), the samples could be well grouped into two clusters, indicating huge transcriptional changes in silicosis (Figure 1A). Differential expression analysis revealed 1,329 differentially expressed genes between the two groups (adjusted p < 0.05; absolute fold change > 2; FPKM ≥ 1 in at least one sample). Among them, 556 genes were up-regulated and 773 genes were down-regulated in the lungs of silicosis patients (Figure 1B).

To elucidate which pathways are predominant in silicosis formation, we next conducted functional enrichment analysis for the differential genes. Items related to “immunity”, “metabolism”, “signaling transduction” and “protein digestion and absorption” were significantly enriched in the KEGG enrichment results under the cutoff of p < 0.05 (Figure 1C and Figure S1A). Consistent with previous studies, the activation of several inflammatory cells, including lymphocytes and neutrophils, were revealed in silicosis lungs (Figure 1C and Figure S1). Notably, several metabolic pathways, including AA metabolism, were highly enriched among the KEGG results, indicating that metabolic reprogramming is a likely step in the development of silicosis (Figure 1C).

### Time-course RNA-seq analysis reveals metabolic reprogramming accompanied with the development of silicosis in mouse lungs

Since the data from human samples only reflect the changes in advanced silicosis, we next constructed a silica-induced silicosis mouse model to characterize the molecular changes that cover the full time span of the silicosis progression. Considering the majority of silicosis patients are male, only male mice were selected for this study. Three weeks, six weeks, and nine weeks after instillation of silica / PBS, we measured the body weight and pulmonary function of mice, and then evaluated their inflammatory status and degree of pulmonary fibrosis. The results showed that, pulmonary dysfunction and pulmonary fibrosis occurred after 3 weeks of silica instillation (Figure S2-4). And with the extended silica exposure time, progression of silicosis was characterized by gradually aggravated fibrosis accompanied with chronic inflammation (Figure S2-4).

**Figure 2:**
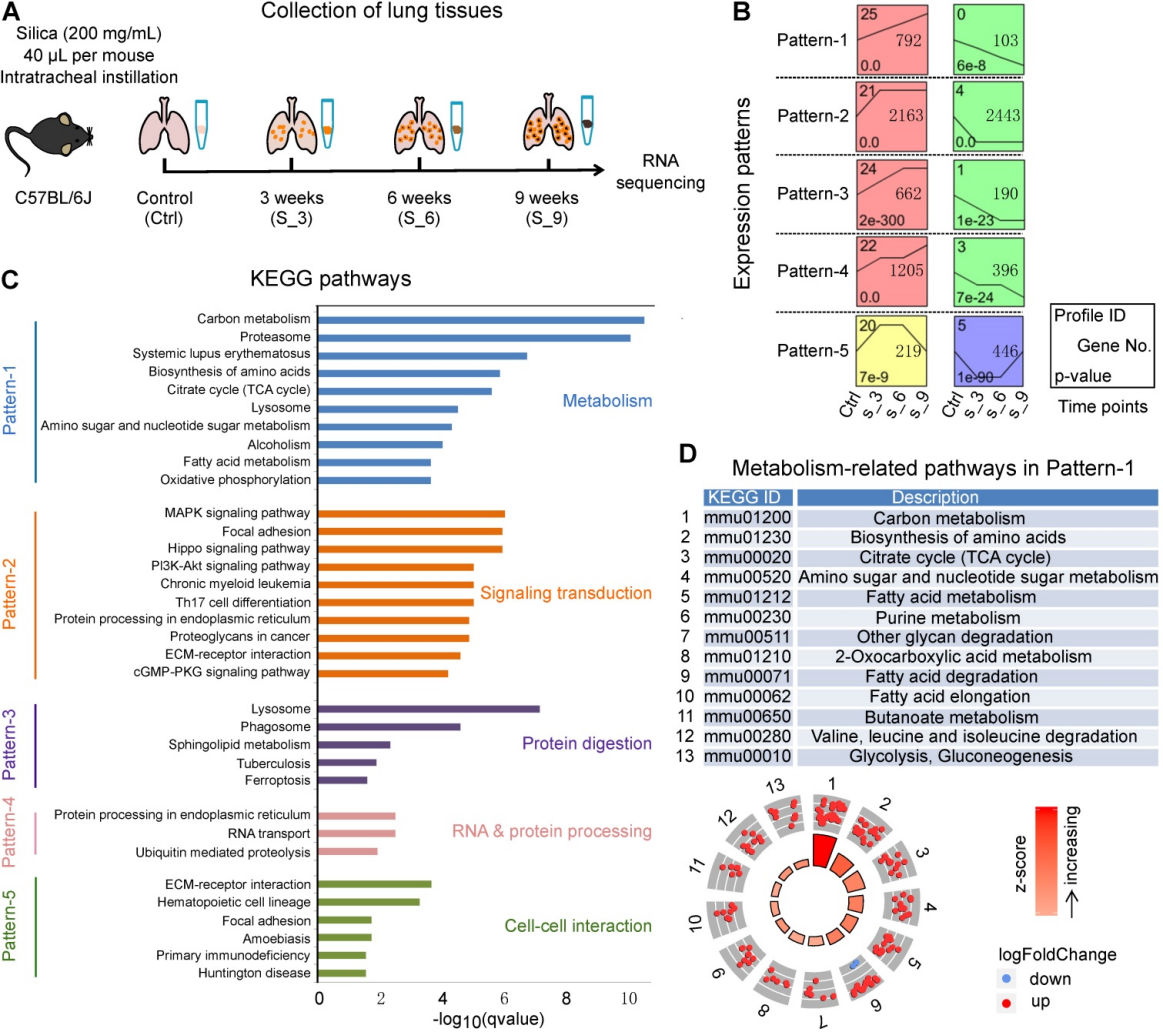
A time-course transcriptional analysis using mouse lungs from silicosis models. (A) Sketch map of the time points to collect lung tissues for RNA sequencing. Mice instilled with PBS for 3 weeks were used as control. Lung tissues from control (Ctrl) along with the silica group at 3, 6 and 9 weeks were collected for RNA sequencing. (B) Significant gene expression patterns identified by the STEM method. The x-axis represents the time points during the model construction, and all the expression patterns follow the lines of Control (Ctrl), Silica 3 weeks (S_3), Silica 6 weeks (S_6) and Silica 9 weeks (S_9). (C) The KEGG enrichment results of the five patterns in (B). For each pattern, only the top ten (if there were) significant items were shown, and five different colors represent the five patterns separately. (D) All the metabolism-related KEGG pathways enriched for Pattern-1. The dots in the outer circle represent the genes in each item, of which the red color highlight the genes up-regulated in silicosis while the blue down-regulated genes. The color bars in the center circle display the z-score, a crude measure of genes' activity in each item. The higher z-score means more up-regulated genes in that item. STEM, short time-series expression miner; KEGG, kyoto encyclopedia of genes and genomes.

To investigate the mechanisms underlying silicosis development, we conducted a time-course RNA-seq analysis using the silica-induced mouse model (Figure 2A). Since there were no significant differences among the three PBS groups (3 weeks, 6 weeks, and 9 weeks) for all the indicators we tested, we thus used mice instilled with PBS for 3 weeks as control. Hierarchical clustering analysis showed that samples collected from control (Ctrl) versus silica-induced mice collected at 3, 6, and 9 weeks of exposure (S_3, S_6, S_9), all grouped individually (Figure S5A). A total of 13,787 differentially expressed protein-coding genes (adjusted p < 0.05) were revealed among the four time points (Ctrl, S_3, S_6, S_9) (Table S2). By setting at least 2 fold expression changes between the maximum and minimum, and with 1,000 permutations per gene, ten significant time-course profiles were revealed under the significance level of 0.05 by Bonferroni correction method (Figure S5B). The ten profiles were then organized into five patterns (Pattern-1 to Pattern-5) according to the times at which they were differentially up- or down-regulated (Figure 2B). Pattern-1 reflects continuously responsive genes that consistently increase or decrease during the disease progression. Pattern-2 contains early-stage responsive genes, which were enhanced or reduced after 3 weeks of silica instillation. Similarly, Pattern-3 contains early and middle-stage responsive genes; Pattern-4 contains early and late-stage responsive genes; and Pattern-5 contains early and late-stage genes with reversed expression.

Further KEGG enrichment analysis showed that the predominant terms in each pattern were associated with diverse pathways, including “metabolism”, “signaling transduction”, “protein digestion”, “RNA & protein processing”, and “cell-cell interaction” (Figure 2C). More importantly, Pattern-1, which contains genes with enduring changes throughout the progression of silicosis, was mainly enriched for metabolism-related pathways. We then checked the gene expression trends in these metabolism-related pathways and found that the majorities were up-regulated after silica treatment, indicating a strong metabolic activation in the progression of silicosis (Figure 2D). Taken together with our findings that differentially expressed genes in human silicosis patients were also significantly enriched in metabolic pathways (Figure 1C), we hypothesized that metabolic reprogramming accompanied the progression of silicosis.

### Untargeted metabolomics shows overall enhancement of the arachidonic acid metabolism in silicosis mouse lungs

**Figure 3:**
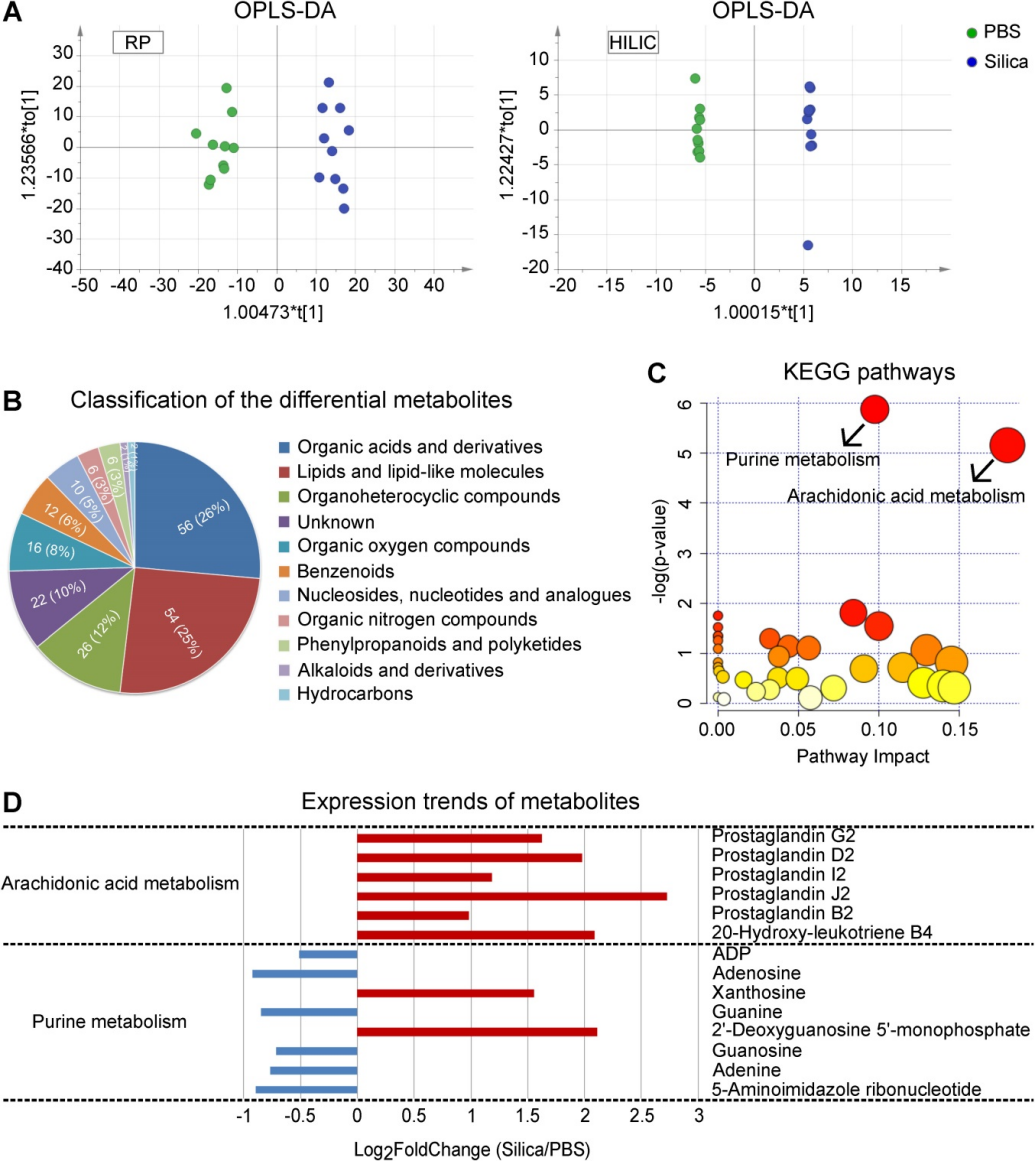
LC-MS-based untargeted metabolomics using silicosis mouse lungs. (A) OPLS-DA of the samples. The green color represents the PBS group (n = 10), while the blue color Silica group (n = 10). Compounds that were selected through RP and HILIC were analyzed separately. (B) Classification of the 212 significantly altered metabolites between PBS and Silica groups. Each color displays a class of metabolites, with the specific number and percentage highlighted in the middle of the pie. (C) KEGG pathway enrichment of the 212 differential metabolites. The two significant pathways with a p < 0.05 were highlighted with their names. (D) The expression trends of the metabolites included in the two significant pathways. The x-axis shows the log_2_ transformed fold change of the relative metabolite level in the Silica group compared to the PBS group. LC-MS, liquid chromatography-tandem mass spectrometry; OPLS-DA, orthogonal partial least squares discriminant analysis; PBS, phosphate-buffered saline; RP, reversed-phase; HILIC, hydrophilic interaction liquid chromatography; KEGG, kyoto encyclopedia of genes and genomes.

In order to identify metabolic changes associated with silicosis, we conducted a comprehensive liquid chromatography-tandem mass spectrometry (LC-MS) based untargeted metabolomic profiling using lung tissues from both silicosis mice and PBS control mice. Both Orthogonal Partial Least Squares Discriminant Analysis (OPLS-DA) and PCA showed wide metabolic differences between these two groups (Figure 3A and Figure S6). Quantitation and identification of metabolites led to identification of 212 differential metabolites, of which 138 were found in higher levels and 74 with lower levels in silicosis compared with control (Table S3).

We next organized the 212 differential metabolites into several super classes, and found that “Organic acids and derivatives” as well as “Lipids and lipid-like molecules” accounted for more than half of the metabolites (Figure 3B), with significant enrichment for “Arachidonic acid metabolism” and “Purine metabolism” (Figure 3C). Furthermore, expression of the AA pathway metabolites showed overall enhancement (Figure 3D), suggesting the critical role of AA metabolism in the progression of silicosis.

### Targeted metabolomics reveals increased expression of PGD_2_, PGE_2,_ and TXA_2_ in silicosis mouse lungs

To confirm the changes in accumulation of AA pathway products, a panel of 106 AA-derived metabolites (Table S4) was quantified using targeted metabolomics. After filtering the metabolites with low concentrations, 47 metabolites above the minimum detection threshold were retained for further statistical analysis (Table S5). Eventually, 16 metabolites were found to be significantly altered (fold change > 2; student's *t*-test p < 0.05) in the lungs of silicosis mice compared to the controls (Table S6).

**Figure 4:**
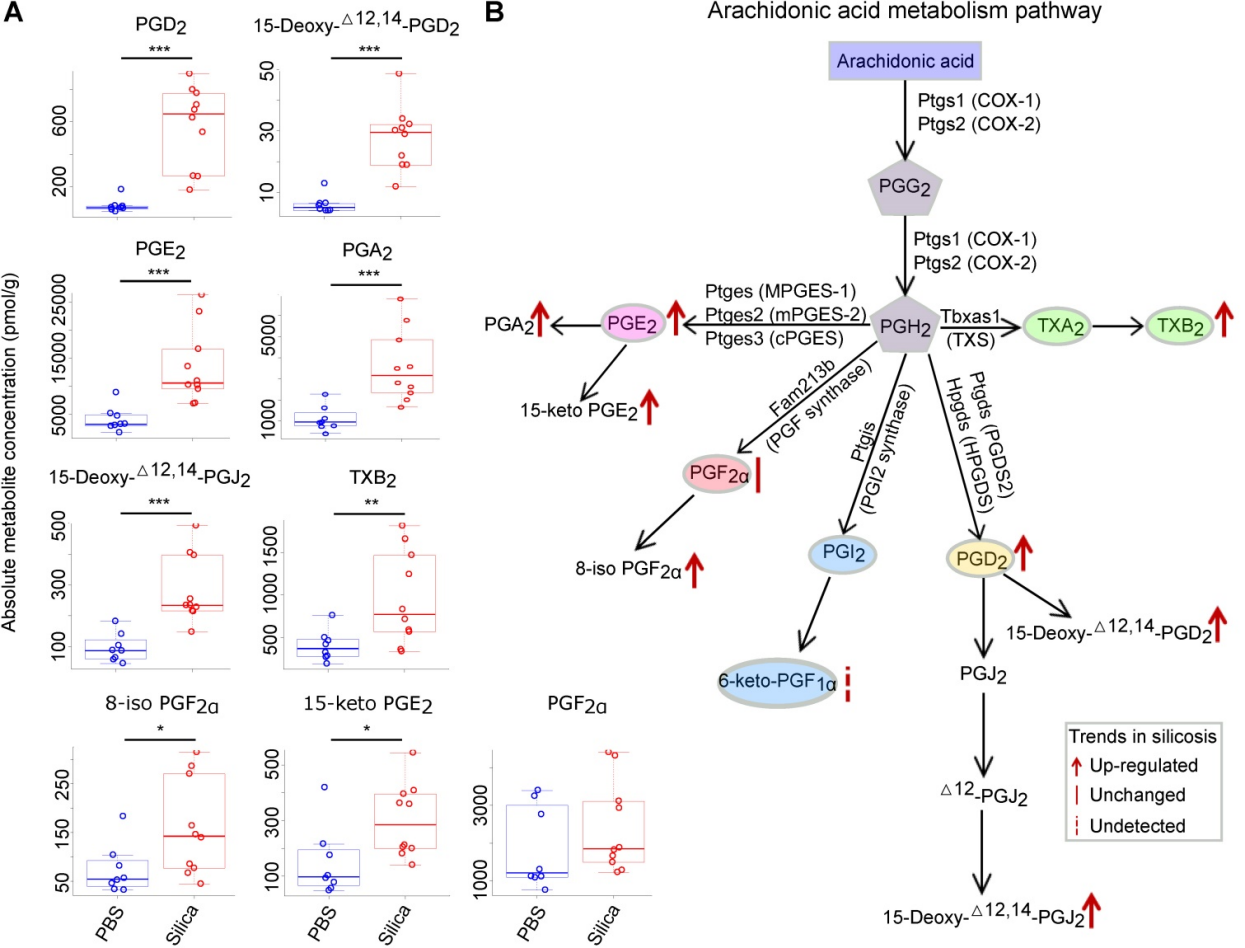
Targeted metabolomics analysis of the arachidonic acid metabolism pathway using silicosis mouse lungs. (A) The absolute metabolite concentration of prostanoids and derivates in both PBS and Silica groups. The comparison between groups was analyzed using the two-tailed unpaired Student's *t*-test. *: p < 0.05; **: p < 0.01; ***: p < 0.001 are representative for the differences of the Silica group (n = 10) as compared to the PBS group (n = 8). (B) The diagram of arachidonic acid metabolism pathway and the metabolic changes in silicosis. PBS, phosphate-buffered saline.

Among the 16 significantly altered metabolites, 11 belonged to prostanoids and their derivates, which was an overrepresented group among all the metabolites tested (p = 0.05, chi-squared test) (Table S6). We thus focused our analysis on the prostanoids, which are generated from AA by cyclooxygenase (COX) (Figure 4B). Among the five prostanoids (PGE_2_, PGF_2α_, PGI_2_, PGD_2_, TXA_2_), PGD_2_, PGE_2_, and TXA_2_ (measured by its stable form TXB_2_) were significantly increased in silicosis mouse lungs (Figure 4A and 4B).

### PGD_2_ and TXA_2_ synthases are significantly up-regulated in human and mouse silicosis lungs

**Figure 5:**
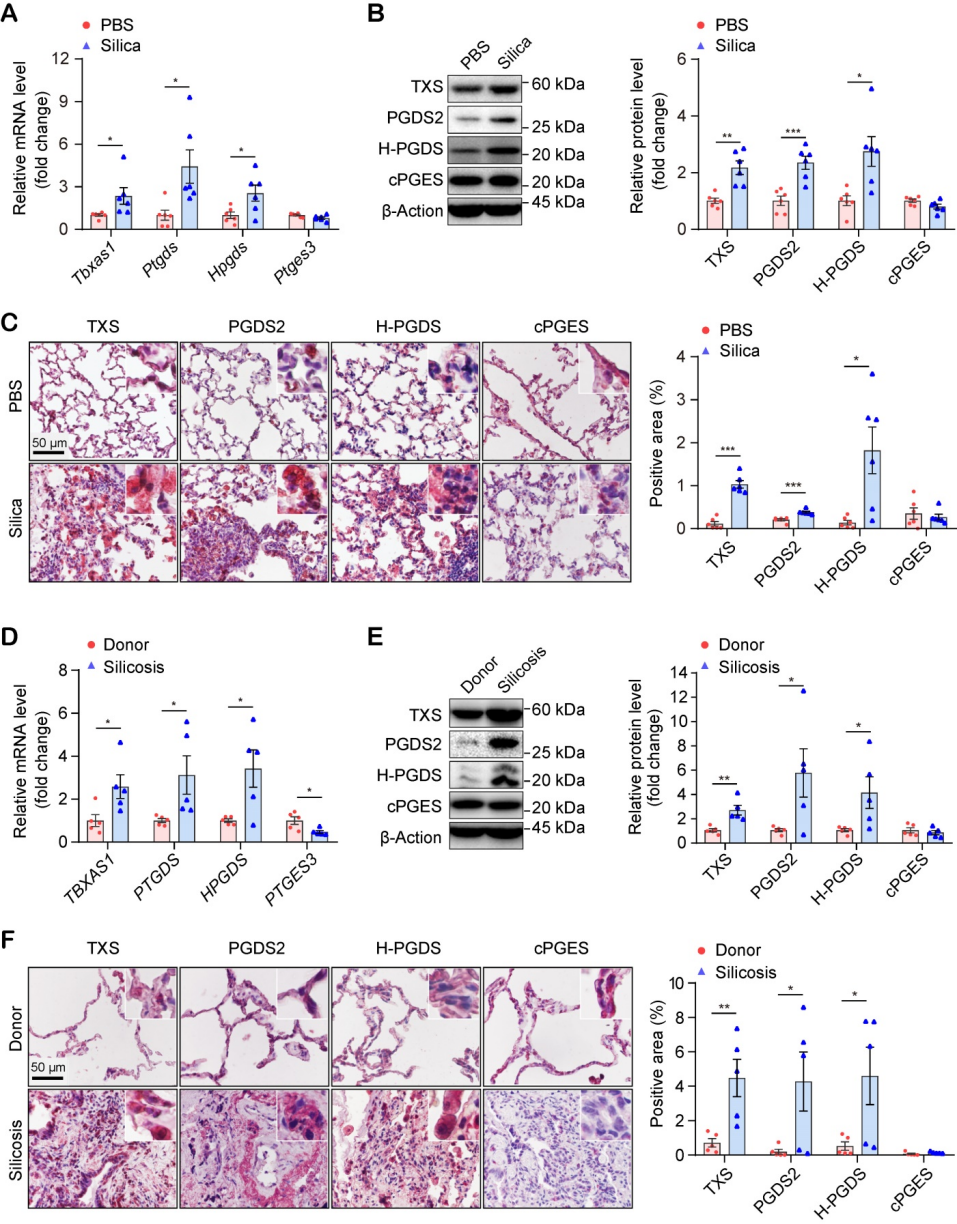
Expressional validation of the synthases of PGD_2_ and TXA_2_ in the lungs of silicosis mice and human patients. (A) Relative mRNA expression levels of the genes (*Tbxas1, Ptgds, Hpgds,* and *Ptges3*) in the lung tissues of mice instilled with silica or PBS. (B) Representative western blotting images and quantification of proteins (TXS, PGDS2, H-PGDS, and cPGES) in the lung tissues of mice instilled with silica or PBS. (C) Representative images of immunohistochemical staining and quantification of immunoreactivity of TXS, PGDS2, H-PGDS, and cPGES in lung sections from the Silica or PBS group. Red-brown indicates the positive staining. All scale bars are 50 μm. (D) Relative mRNA expression levels of the genes (*TBXAS1, PTGDS, HPGDS,* and *PTGES3*) in the lung tissues from silicosis patients or healthy donors. (E) Representative western blotting images and quantification of protein (TXS, PGDS2, H-PGDS, and cPGES) in lung tissues from silicosis patients or healthy donors. (F) Representative images of immunohistochemical staining and quantification of immunoreactivity of TXS, PGDS2, H-PGDS, and cPGES in lung sections from silicosis patients or healthy donors. Red-brown indicates the positive staining. All scale bars are 50 μm. All the above experiments were performed at least three times. Quantitative analysis results are presented as mean ± SEM, and the differences were analyzed by using the two-tailed unpaired Student's* t-*test. *: p < 0.05, **: p < 0.01, and ***: p < 0.001 in A, B, C are representative for the differences from the Silica group (n = 6 each group) *versus* the PBS group (n = 6 each group), and in D, E, F are representative for the differences from the Silicosis group (n = 5 each group) *versus* the donor group (n = 5 each group). PGD_2_, prostaglandin D_2_; TXA_2_, thromboxane A_2_; PBS, phosphate-buffered saline.

To validate the results of the targeted metabolomic analysis, we checked the mRNA and protein levels of the genes encoding the critical synthases of TXA_2_ (*Tbxas1* / TXS), PGD_2_ (*Ptgds* / PGDS2 and *Hpgds* / H-PGDS), and PGE_2_ (*Ptges3* / cPGES), in mouse lungs. As shown in Figure 5A, the mRNA levels of the genes encoding the synthases of TXA_2_ and PGD_2_, but not PGE_2_, were significantly up-regulated in silicosis mouse lungs. Similarly, the corresponding protein levels (TXS, PGDS2, and H-PGDS) of these three genes were also significantly up-regulated in silicosis mouse lungs detected by both western-blot (Figure 5B) and immunohistochemistry (IHC) staining (Figure 5C and Figure S7).

We further examined the expression levels of these synthases in human lungs. Consistent with the results in mice and in support of the RNA-seq results, the both mRNA and protein expression of PGD_2_ synthases and TXA_2_ synthase were also significantly increased in the lungs of silicosis patients compared with controls (Figure 5D-F and Figure S8, S9). Taken together, these data suggested that PGD_2_ and TXA_2_ may participate in the pathogenesis of silicosis.

### Ramatroban, as an antagonist of PGD_2_ and TXA_2_ receptors, inhibits silicosis progression

It is well known that increased levels of TXA_2_ and PGD_2_ can enhance the interaction with their respective receptors, *i.e.,* thromboxane prostanoid (TP) receptor and PGD_2_ receptors including D-prostanoid receptor 1 (DP1), DP receptor 2 (DP2), and TP receptor. Ramatroban, as a selective blocker of the DP2 and TP receptors 26, can thus inhibit the proinflammatory, profibrotic, and vasoconstriction effects mediated by the PGD_2_-DP2 and TXA_2_/PGD_2_-TP receptor axes 27, 28. Therefore, we hypothesized that Ramatroban may also alleviate the progression of silicosis. In actual clinical situations, silicosis patients are frequently admitted in the fibrotic stage due to the difficulty of correct diagnosis. Our preliminary results showed that initiation of Ramatroban treatment in mice was appropriate after three weeks of silica instillation to most accurately simulate the clinical conditions in humans (Figure S2-4).

**Figure 6:**
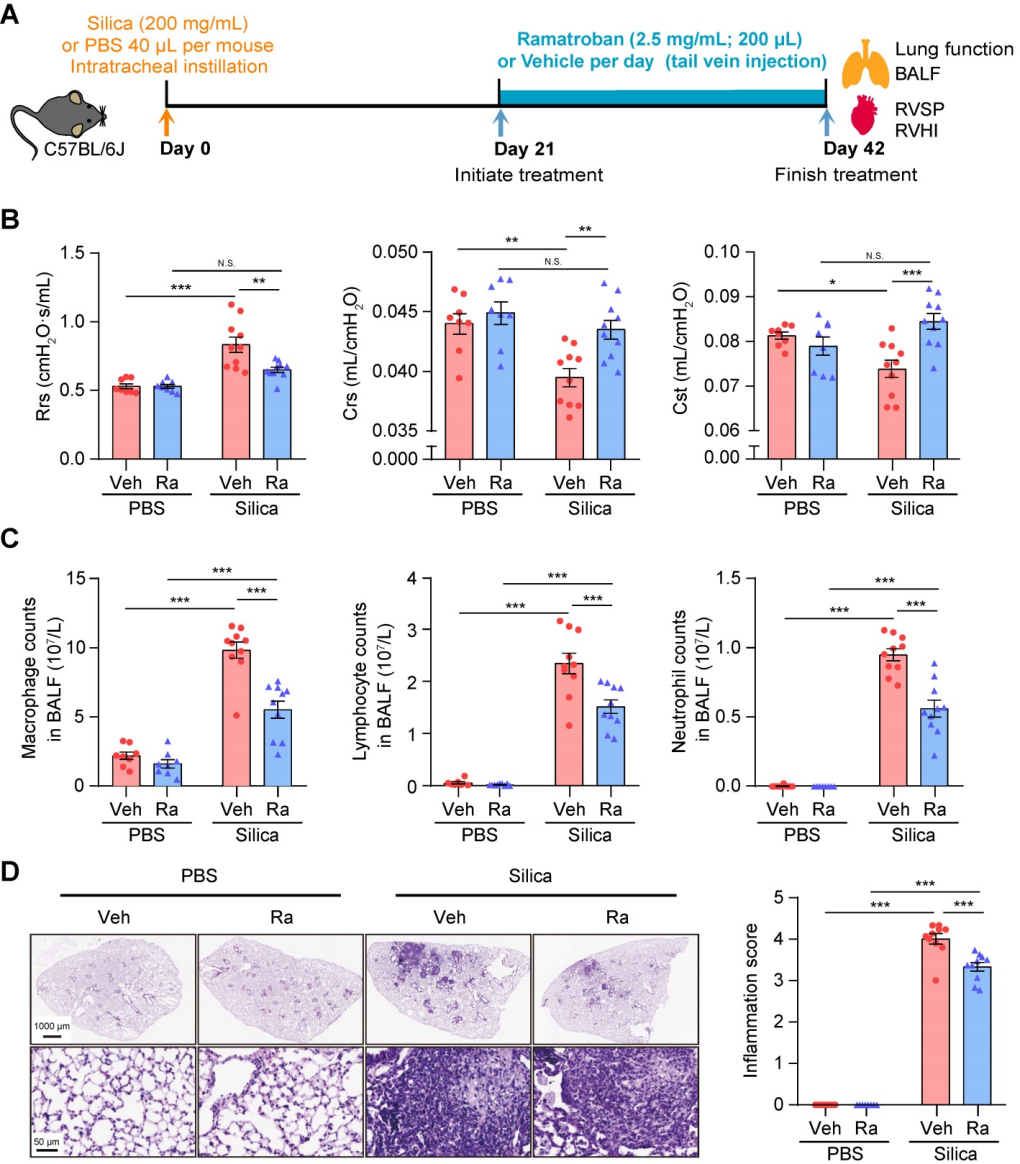
Ramatroban treatment improved pulmonary function and inflammation in silicosis mice. (A) Schematic diagram of Ramatroban treatment on silicosis mice. (B) Measurements of parameters of pulmonary function, including Rrs, Crs, and Cst. (C) Counts of inflammatory cells (macrophages, lymphocytes, and neutrophils) in BALF. (D) Representative images of hematoxylin-eosin staining and quantification of pulmonary inflammation. Upper scale bar indicates 1000 μm, and lower scale bar indicates 50 μm. All the above experiments were performed at least three times. All the quantitative results are presented as mean ± SEM. The differences were analyzed by a two-way ANOVA and followed by Bonferroni adjustment. N.S.: no significance; *: p < 0.05, **: p < 0.01, and ***: p < 0.001. PBS group: n = 8 each group; Silica group: n = 10 each group. PBS, phosphate-buffered saline; BALF, bronchoalveolar lavage fluid; RVSP, right ventricular systolic pressure; RVHI, right ventricular hypertrophy index; Veh: vehicle; Ra: Ramatroban; Rrs, resistance; Crs, compliance of the respiratory system; Cst, quasi-static lung compliance.

All mice survived until they were sacrificed at 3 weeks post-treatment (Figure 6A), and no organ toxicity was observed in Ramatroban-treated mice (Figure S10). As shown in Figure 6B and Figure S11, Ramatroban significantly relieved the impairment of pulmonary function, and alleviated abnormal right ventricular systolic pressure (RVSP), right ventricular hypertrophy index (RVHI), as well as pulmonary artery remodeling induced by silica. The silica-induced increase of inflammatory cells in both bronchoalveolar lavage fluid (BALF) and lung tissues was remarkably reduced by Ramatroban (Figure 6C and 6D). Moreover, proinflammatory cytokines including TNF-α, IL-6, IL-1β, and IL-18 in supernatants from BALF were significantly reduced in Ramatroban-treated mice compared with control mice (Figure S12). Since it has been widely reported that IL-1β and IL-18 are produced by Nucleotide-binding domain like receptor protein 3 (NLRP3) inflammasome through activating caspase-1 29, 30, we further assessed the expression of NLRP3, caspase-1, and IL-1β in lung tissues. Our data showed that the expression of NLRP3, caspase-1, and IL-1β, at both the mRNA and protein levels, were significantly increased in response to silica stimulation and were significantly reduced by Ramatroban treatment (Figure S13).

**Figure 7:**
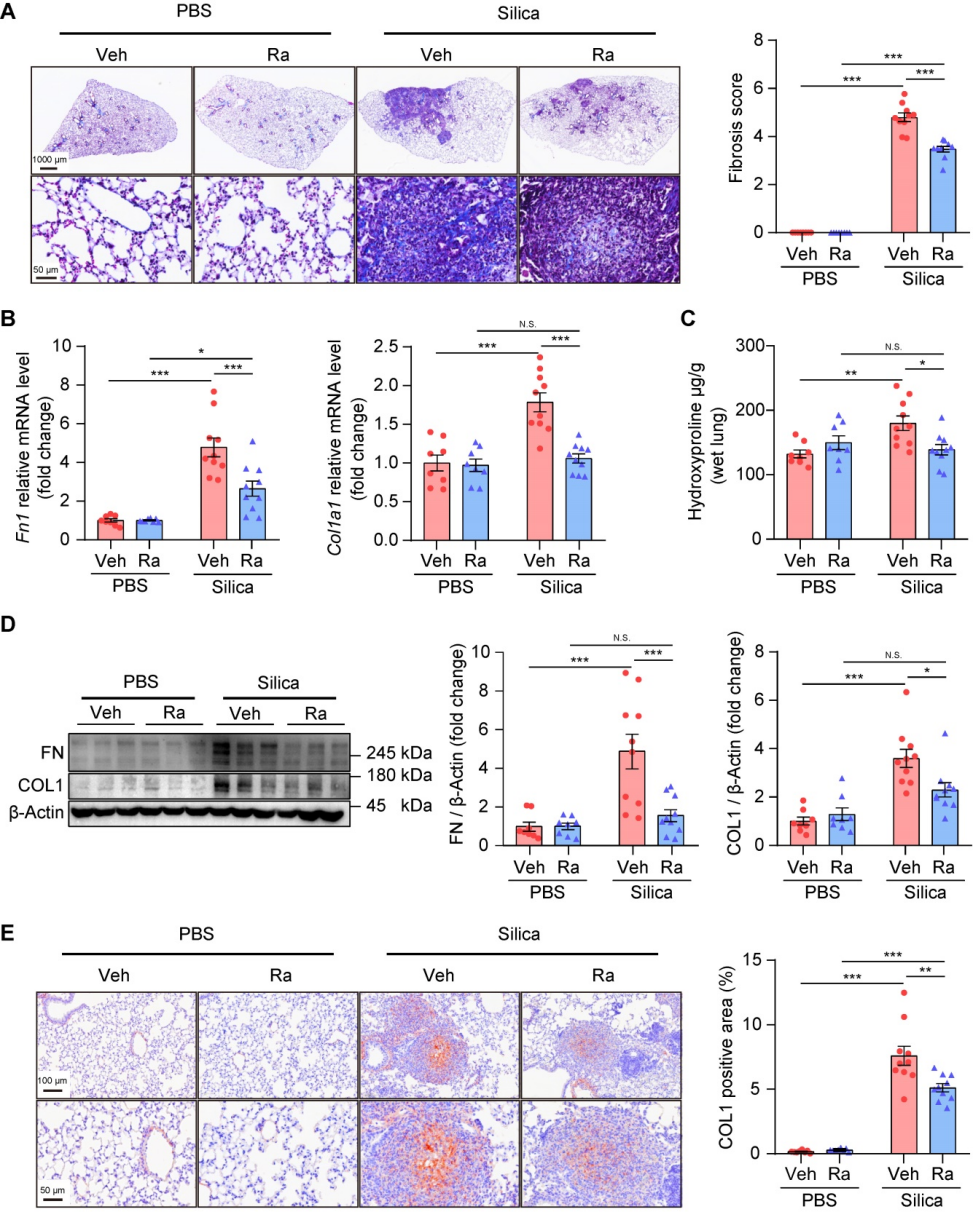
Ramatroban treatment alleviated pulmonary fibrosis in silicosis mice. (A) Representative images of Masson staining and quantification of pulmonary fibrosis. Upper scale bar indicates 1000 μm, while lower scale bar indicates 50 μm. (B) Relative mRNA expression levels of *Fn1* and *Col1a1*. (C) Hydroxyproline levels evaluated from mouse lungs. (D) Representative western blotting images and quantification of FN and COL1. (E) Representative images of immunohistochemical staining and quantification of immunoreactivity of COL1 in lung sections. Red-brown indicates the positive staining. Upper scale bar indicates 100 μm, and lower scale bar indicates 50 μm. All the above experiments were performed at least three times. All the quantitative results are shown as mean ± SEM. The differences were calculated by a two-way ANOVA and followed by Bonferroni adjustment. N.S.: no significance; *: p < 0.05, **: p < 0.01, and ***: p < 0.001. Mouse lung tissues are from the Silica group (n = 10) or the PBS group (n = 8) following Ramatroban (Ra) or vehicle (Veh) treatment. Fn or FN, fibronectin; Col or COL, collagen; PBS, phosphate-buffered saline.

In addition, Ramatroban significantly attenuated lung fibrosis, indicated by decreased collagen fiber content (Figure 7A), reduced fibrosis score (Figure 7A), decreased hydroxyproline level (Figure 7C), and down-regulated expression of fibronectin and collagen I at both the mRNA (Figure 7B) and protein levels (Figure 7D-E and Figure S14). Together, these results suggested that Ramatroban may serve as a therapeutic agent to prevent the progression of silicosis through anti-inflammatory and anti-fibrotic properties, as well as cardiopulmonary protective effects, without producing obvious toxicity.

## Discussion

In the present study, we integrated multi-omics approaches to reveal the molecular changes in silicosis lungs. RNA-seq analysis in the lungs of silicosis patients and mice showed that metabolic reprogramming accompanied the progression of silicosis. Using a combination of untargeted and targeted metabolomic analyses, the AA metabolic pathway was found to participate in silicosis progression. Further validation of the mRNA and protein levels of AA metabolite synthases revealed the consistent up-regulation of two prostanoids, PGD_2_ and TXA_2_, in silicosis lungs, indicating that PGD_2_ and TXA_2_ may participate in the pathogenesis of silicosis. We subsequently confirmed that Ramatroban, an antagonist of PGD_2_ and TXA_2_ receptors, could successfully alleviate the progression of silicosis in a mouse model, indicating its potential clinical application for treating silicosis.

PGD_2_ and TXA_2_ have important roles in the processes of inflammation and fibrogenesis. TXA_2_ promotes allergic inflammation 31 and pulmonary fibrosis 32, while the role of PGD_2_ in inflammation and fibrogenesis remains controversial. In some studies, the increased level of PGD_2_ was suggested to have proinflammatory 31 or profibrotic 33 roles in disease development. In contrast, other studies have reported its anti-inflammatory role in acute lung injury 34 or anti-fibrotic role in pulmonary fibrosis 35, 36. In silica-induced pulmonary fibrosis, AMs have been demonstrated to produce and release PGD_2_ and TXA_2_ when exposed to silica 37-39. However, their *in vivo* roles in human and animal silicosis models are still unclear. Our study found, using an integrated multi-omics approach with experimental validation, that PGD_2_ and TXA_2_ are significantly increased during silicosis. A subsequent drug test using Ramatroban, through antagonizing the receptors of PGD_2_ and TXA_2_, further demonstrated that PGD_2_ and TXA_2_ are crucial for promoting pulmonary inflammation and fibrosis in silicosis.

Ramatroban is a clinically available drug that has been approved for the treatment of allergic rhinitis 40. Previous studies mainly focused on its anti-inflammatory effects in asthma and allergic diseases 41. Our results demonstrated that Ramatroban also has anti-fibrotic and cardiopulmonary protective effects, which may expand its clinical indications.

Prior to our research, several studies explored the potential usage of clinically available drugs in treating silicosis using animal models. Guo et al. reported that pirfenidone could reduce the silica-induced alveolar inflammation and fibrosis in rat 42. Other reports have shown that Baicalin 43, Dioscin 44, and Dihydrotanshinone I 45 can alleviate silica-induced inflammation and/or fibrosis in mice. Similarly, our results showed remarkable alleviation of pulmonary inflammation and fibrosis in silicosis mice treated with Ramatroban. In contrast to these previous studies in which drugs were mainly given one day after silica instillation, we began Ramatroban treatment after three weeks of silica exposure when the mouse model had developed to a fibrotic stage. Given that the treatment for silicosis patients is usually in the fibrotic stage due to the difficulty of clinical diagnosis, our study suggested that Ramatroban may be an effective anti-fibrotic agent for silicosis patients. Additionally, our study also showed that Ramatroban attenuated pulmonary vascular remodeling and had a protective effect on cardiovascular function in silicosis mice. Notably, pulmonary hypertension (PH) is among the leading causes of death for silicosis patients 46. We therefore suggest further exploration of Ramatroban as a candidate drug to treat silicosis patients complicated with PH.

The mechanisms of how PGD_2_ and TXA_2_ were increased and involved in the development of silicosis still lack elaboration. Previous reports suggested that miRNAs may be involved in the generation and down-stream signaling transduction of PGD_2_ and TXA_2_. The expression of miR-520 was conversely correlated with that of COX and PTGDS of the PGD_2_ synthesis pathway in patients with vessel restenosis 47. Over-expression of miR-20a can reduce the expression level of TXA_2_ in human umbilical vein endothelial cells 48. In addition, miR-592-5p may inhibit the PGD_2_/DP signaling pathway to protect against hippocampal neuronal injury 49. In silicosis, a growing number of studies have either demonstrated the critical anti-fibrotic 50 or pro-fibrotic 51 roles of miRNAs, or suggested the potential usage of miRNAs as diagnostic biomarkers for pulmonary fibrosis 52. All the above reports suggest that some miRNAs may function upstream of the generation of PGD_2_ and TXA_2_, or in the process of PGD_2_ and TXA_2_ transduction, thus contributing to the progression of silicosis. Further studies are worthwhile to detect the crosstalk between miRNAs and the pathways generating or acting in transduction of PGD_2_ and TXA_2_ in silicosis.

Previous investigations have established that silica exposure can lead to oxidative stress and excessive accumulation of reactive oxygen species (ROS), followed by the activation of ROS-NLRP3 signaling in AMs 29, 30. The expression of its downstream factors including caspase-1, IL-1β, and IL-18 was then increased, which contributed to silica-induced pulmonary inflammation and fibrosis 29, 30. A recent study showed that TXA_2_ activates the TP receptor, leading to oxidative injury via enhanced nicotinamide adenine dinucleotide phosphate (NADPH) oxidase activity and ROS generation 53. Similarly, Rossi et al. suggested that PGD_2_ promotes ROS generation 54. These findings suggest that PGD_2_ and TXA_2_ may promote silicosis progression through the ROS-NLRP3 signaling pathway. Consistent with these findings, we observed silica-induced increases in the expression of NLRP3, caspase-1, IL-1β, and IL-18. Moreover, their expression levels can be reduced by treatment with Ramatroban, an antagonist of PGD_2_ and TXA_2_ receptors (Figure S13). Thus, we speculated that Ramatroban may exert its anti-inflammatory and anti-fibrotic effects mediated by down-regulating ROS-NLRP3 signaling. Furthermore, there could be other potential mechanisms of Ramatroban in alleviating silicosis progression. Currently, increased PGD_2_ and TXA_2_ levels have been demonstrated to promote the production of proinflammatory cytokines (*e.g.* IL-4, IL-5, and leukotriene B4 (LTB4), etc.) and profibrotic cytokines (*e.g.* IL-10, IL-13, and TGF-β, etc.) mediated by activating DP2 and TP receptors 27, 28, 31, 32, 55. Simultaneously, blocking DP2 or TP receptor can attenuate pulmonary inflammation and fibrosis in fibrotic diseases 28, 56, 57. Based on these findings, we proposed that another potential mechanism of Ramatroban's protective actions is mediated by down-regulating the expression of some cytokines such as IL-4 and IL-5. Further studies are needed to evaluate the expression changes of these cytokines.

We acknowledge some limitations of this study. First, the underlying mechanisms of Ramatroban alleviating silicosis development still need to be addressed in further studies. Second, since the dose for Ramatroban treatment in silicosis mice was converted from the commonly used clinical dose of allergic rhinitis, the optimal dose of Ramatroban used for silicosis needs further exploration. Third, only a limited amount of human lung tissues was acquired for this study, meaning that metabolomics studies could not be performed in these samples. The use of human samples for metabolomics studies would provide sound validation of our evidence obtained from the mouse model. In summary, our study revealed that PGD_2_ and TXA_2_ participate in the progression of silicosis through the integration of transcriptomics with untargeted and targeted metabolomics analyses. As a dual antagonist for receptors of PGD_2_ and TXA_2_, Ramatroban alleviated silicosis in a silica-induced mouse model, which provides potential therapeutic targets and valuable insights for the treatment of this disease.

## Supplementary Material

Supplementary experimental procedures, figures.

Supplementary tables.

## Abbreviations

AA: arachidonic acid
PGD_2_: prostaglandin D_2_
TXA_2_: thromboxane A_2_
AM: alveolar macrophage
IL-6: interleukin-6
IL-1β: interleukin-1 beta
TNF-α: tumor necrosis factor-alpha
TGF-β: transforming growth factor-beta
IGF-1: insulin-like growth factor-1
PBS: phosphate-buffered saline
RNA-seq: RNA sequencing
FPKM: Fragments per Kilobase Million
STEM: Short Time-series Expression Miner
GO: Gene Ontology
KEGG: Kyoto Encyclopedia of Genes and Genomes
SEM: standard error of the mean
PCA: principal component analysis
HE: hematoxylin-eosin
LC-MS: liquid chromatography-tandem mass spectrometry
OPLS-DA: Orthogonal Partial Least Squares Discriminant Analysis
COX: cyclooxygenase
IHC: immunohistochemistry
TP: thromboxane prostanoid
RVSP: right ventricular systolic pressure
RVHI: right ventricular hypertrophy index
BALF: bronchoalveolar lavage fluid
IPF: idiopathic pulmonary fibrosis
DP: D-prostanoid
NLRP3: nucleotide-binding domain like receptor protein 3
HPS: Hermansky-Pudlak syndrome
ROS: reactive oxygen species
NADPH: nicotinamide adenine dinucleotide phosphate
PH: pulmonary hypertension
Th2: T-helper lymphocyte type-2
LTB4: leukotriene B4
