# Supplementary material for: Multi-omics study of silicosis reveals the potential therapeutic targets PGD2 and TXA2
Source: Theranostics. 2021 Jan 1;11(5):2381–94. doi: 10.7150/thno.47627 (PMC7797695; doi:10.7150/thno.47627)
Supplement: Supplementary file 1 — Supplementary experimental procedures, figures. [file thnov11p2381s1.pdf]

Multi-omics study of silicosis reveals the potential therapeutic targets PGD_2 and TXA_2

Junling Pang*, Xianmei Qi*, Ya Luo*, Xiaona Li, Ting Shu, Baicun Li, Meiyue Song, Ying Liu, Dong Wei, Jingyu Chen†, Jing Wang†, Chen Wang

Supplementary Experimental Procedures

RNA sequencing

1) RNA qualification

The quality of the RNA samples was firstly evaluated before performing downstream experiments. The RNA degradation and contamination was monitored through 1% agarose gels visualization. RNA purity was checked using the NanoPhotometer® spectrophotometer (IMPLEN, CA, USA). RNA concentration was measured using the Qubit® RNA Assay Kit in Qubit®2.0 Flurometer (Life Technologies, CA, USA). RNA integrity was assessed using the RNA Nano 6000 Assay Kit of the Bioanalyzer 2100 system (Agilent Technologies, CA, USA).

2) Library preparation

A total of 3 μg of RNA per sample was used as input for the RNA sample preparation. Sequencing libraries were generated using NEBNext® Ultra™ RNA Library Prep Kit for Illumina® (NEB, USA) following the manufacturer's recommendations and index codes were included to infer sequences from each sample. Briefly, mRNA was purified from total RNA using poly-T oligo-attached magnetic beads. Fragmentation was carried out using divalent cations under elevated temperature in NEBNext First Strand Synthesis Reaction Buffer (5X). First strand cDNA was synthesized using random hexamer primer and M-MuLV Reverse Transcriptase (RNase H-). Second strand cDNA synthesis was subsequently performed using DNA Polymerase I and RNase H. Remaining overhangs were converted into blunt ends via exonuclease/polymerase activities. After adenylation of the 3' ends of DNA fragments, NEBNext Adaptor with hairpin loop structures were ligated for hybridization. In order to select cDNA fragments of preferentially 250-300 bp in length, the library fragments were purified with an AMPure XP system (Beckman Coulter, Beverly, USA). Then 3 μL USER Enzyme (NEB, USA) was used with size-selected, adaptor-ligated cDNA at 37 °C for 15 min followed by 5 min at 95 °C before polymerase chain reaction (PCR). Then PCR was performed using a Phusion High-Fidelity DNA polymerase, universal PCR primers and Index (X) Primer. Finally, PCR products were purified (AMPure XP system) and the quality of the library was assessed using an Agilent Bioanalyzer 2100 system.

3) Clustering and sequencing

The clustering of the index-coded samples was performed on a cBot Cluster Generation System using TruSeq PE Cluster Kit v3-cBot-HS (Illumina) according to the manufacturer's instructions. After cluster generation, the library preparations were sequenced on an Illumina Hiseq platform and 150 bp paired-end reads were generated.

4) Quality control

Raw reads of fastq format were firstly processed through in-house perl scripts. In this step, clean reads were obtained by removing reads containing adapter, reads containing poly-N and low quality reads from raw data. In parallel, Q20, Q30 and GC content were calculated on the cleaned data. All the downstream analyses were based on high quality cleaned data.

Silicosis mouse model

Crystalline silica (CAS7631-86-9, Forsman Scientific Co., Ltd, Beijing, China) with a average particle diameter 1.6 μm and purity 99%, was prepared endotoxin-free by heating them at 200 °C for 2 h and was suspended in sterile phosphate-buffered saline (PBS). Suspensions were sonicated for 10 min before use.

Male 8-10 weeks old C57BL/6J mice (22 ± 3 g) were purchased from the Vital River Laboratory Animal Technology Co. Ltd. (Beijing, China). All mice were housed in a specific pathogen-free animal room at 60-70% humidity and 24-26 ℃, with a 12 / 12 h light-dark cycle. We performed an exploring experiment conducted in a silicosis mouse model using a single intratracheal instillation with different crystalline silica dose and exposure time (data not shown). Based on the results from the experiment, the mice were administered with 8 mg of silica in 40 μL sterile PBS by surgically intratracheal instillation to induce silicosis. Briefly, C57BL/6J mice were placed on a platform and then anesthetized with a 2% pentobarbital intraperitoneal injection. Control animals received an equal amount of sterile PBS identical to their counterparts given silica suspensions. The Animal Care and Use Committee of IBMS/PUMC approved all procedures.

Ramatroban treatment

Ramatroban (BAY u3405, Cayman, Ann Arbor, MI, USA) was dissolved in sterile dimethyl sulfoxide (30 mg/mL) and was diluted to 2.5 mg/mL with sterile PBS. Specifically, we fixed the mice first, then heated the tail with warm water to make the veins more visible. Three weeks after silica / PBS instillation, the mice were treated with 200 μL solution of Ramatroban or vehicle via tail vein injection everyday with a 0.3 mL insulin syringe for consecutive 3 weeks [1-3].

Measurements of pulmonary function

Anesthetized mice were subjected to surgical intubation of the trachea with a tracheal cannula, and pulmonary function was measured by a computer-controlled ventilator (FlexiVent, SCIREQ, Montreal, Canada). The parameters of pulmonary function were evaluated by fitting pressure with a forced oscillation test, including inspiratory capacity (IC), resistance (Rrs), compliance of the respiratory system (Crs), and quasi-static lung compliance (Cst).

Measurements of haemodynamic status and hypertrophy index of right ventricle

Right ventricular systolic pressure (RVSP) was measured by closed-chest insertion, with a 22-gauge needle via a xiphocostal angle parallelly inserted into the right ventricle (RV). We identified the position of the needle depending on the detected waveform. RVSP was measured through a connection to a pressure transducer which was interfaced with a Power

Lab Data Acquisition and Analysis System (PL 3504, AD Instruments, Sydney, Australia). The values of RVSP were obtained from the region of the steady waveform.

The right ventricular hypertrophy index (RVHI), a parameter used to evaluate the degree of hypertrophy of RV, was calculated by weighing the RV and left ventricle plus interventricular septum (S). RVHI was calculated by using the following formula RVHI = [RV / (LV + S)].

Measurements of pulmonary arterial media thickness

Collected mouse lung tissues were fixed in 10% phosphate-buffered neutral formalin solution no less than 24 h and then the fixed tissues were dehydrated through xylene and graded alcohol and then embedded in paraffin. Paraffin-embedded tissues were sectioned into 3-5 μm thick sections. Lung tissue sections were stained with Hematoxylin-eosin (HE). To quantitate pulmonary arterial medial wall thickness, the vascular external and internal wall were outlined and perimeter sizes were measure using NDP.view 2. The vascular remodeling was calculated as follows: percentage of media thickness (MT%) = (external wall perimeter / π - internal wall perimeter / π) × 100 / (external wall perimeter / π). At least 40 vessels with an external diameter of below 100 μm per mouse were measured and we calculated the mean values of MT% to assess the remodeling of pulmonary vascular.

Collection of BALF and counts of inflammatory cells

In order to determine the frequency of inflammatory cells (macrophages, lymphocytes, and neutrophils) in the bronchial and alveolar space a bronchoalveolar lavage fluid (BALF) was performed. The lungs were lavaged twice with 0.7 mL PBS via exposed trachea. If necessary, erythrocyte lysis buffer was used to remove red blood cells firstly, and then BALF samples were centrifuged for 5 min at 1500 rpm at 4 °C. The pellet was then resuspended in 300-600 μL sterile PBS and total numbers of inflammatory and epithelial cells were counted using a Neubauer chamber method.

ELISA

Quantification of pro-inflammatory cytokines, including tumor necrosis factor-alpha (TNF-α), interleukin-6 (IL-6), interleukin-1 beta (IL-1β), and IL-18 were performed by enzyme-linked immunosorbent assay (ELISA) using the collected supernatants from BALF. ELISA was performed following the manufacturer's instructions. Further experimental details are listed in the supplementary materials (see online table S7).

LC-MS based untargeted metabolomics analysis

1) Sample preparation

Tissue samples (about 50 mg) were washed using 1 mL of 1X PBS to remove blood from the tissue. Tissue was then washed twice using a 1 mL of pure water to wash off the PBS. 1.2 mL of 80% methanol was then added to the tissue along with beads to lyse it. After 45 s of lysis cycles, the sample was centrifuged at 14000 g for 15 min at 4 ℃, and then dried. The dried sample was then resuspended in 200 uL of 12% acetonitrile solution until all precipitates were dissolved. Centrifugation for 14000 g at 4 ℃ was performed for 10 min after sonication. Centrifuged re-dissolved samples were then applied to a new membrane, and centrifuged at 14000 g at 4 ℃ to make it dry without liquid. Sample was then analyzed by mass spectrometry.

2) LC-MS Analysis

Ultra-performance liquid chromatography-tandem mass spectrometry (LC-MS) analyses of samples were conducted using a Waters ACQUITY H-class LC system coupled with a LTQ-Orbitrap mass spectrometer (Thermo Fisher Scientific, MA. USA). The metabolites were separated with a 18 min gradient on a Waters HSS C18 column (3.0 × 100 mm, 1.7 μm) at a flow rate of 0.5 mL/min. Mobile phase A was 0.1% formic acid in H_2O and mobile phase B was acetonitrile. The gradient was set as it follows: 0-2 min, 2% solvent B; 2-5 min, 2-55% solvent B; 5-13 min, 55-100% solvent B； 13-15 min, 100% solvent B; 15-18 min, 2% solvent B. The column temperature was set at 50 ℃. Full MS acquisition scanned from 100 to 1000 m/z at a resolution of 60 K. Automatic gain control (AGC) target was 1×10^6 and maximum injection time (IT) was 100 ms. UPLC targeted-MS/MS analyses were acquired at a resolution of 15 K with AGC target of 5×10^5, maximum IT of 50 ms, and isolation window of 3 m/z. Collision energy was optimized at 20, 40, 60 or 80 for each target with higher-energy collisional dissociation (HCD) fragmentation. The injection order of the samples with 3 technical replicates was randomized to reduce the experimental bias.

3) Data processing

Raw data files were processed using the Progenesis QI (Waters, Milford, MA, USA) software. Further data pre-processing including missing value estimation, Log transformation and Pareto scaling were carried out to make features more comparable using MetaAnalyst (http://www.metaboanalyst.ca). Variables missed in 50% samples, were removed from further statistical analysis. Pattern recognition analysis (principal component analysis, PCA; orthogonal partial least squares discriminant analysis, OPLS-DA) was performed using the SIMCA 14.0 (Umetrics, Sweden) software.

The preprocessing results generated a data matrix that consisted of the retention time (RT), mass-to-charge ratio (m/z) values, and peak intensity. In-house MS2 database combined with the HMDB database (2017 version) was applied for metabolites identification. Confirmation of the differential compounds was performed by the parameters, including Score, Fragmentation score, and Isotope similarity given by Progenesis QI. A score ranging from 0 to 60 was used to quantify the reliability of each identity. According to the score results of the reference standards, the threshold was set at 35.0.

4) Classification of metabolites

The classification of metabolites was based on the classification of the Human Metabolome Database (HMDB; http://www.hmdb.ca/) as well as using the PubChem database (https://pubchem.ncbi.nlm.nih.gov/).

Targeted metabolomics analysis

1) Reagents

The 106 eicosanoids we tested and their deuterium-labeled internal standards (ISs) (see online table S4) were purchased from Cayman Chemical (Ann Arbor, MI, USA). Formic acid, HPLC grade acetonitrile and methanol were purchased from Thermo Fisher (Thermo Fisher

Scientific Inc, USA), and all other chemicals in this analysis were purchased from Sigma-Aldrich (Sigma-Aldrich, Inc, USA).

2) Sample preparation

Tissue samples (about 50 mg) and 10 μL of BHT (Butylated hydroxytoluene)/MeOH solution (W:V, 4.8 g/100 mL) were subjected to protein precipitation by adding 100 μL of MeOH containing deuterium-labeled internal standards (IS), at a final concentration of 50 ng/mL of the IS. Samples were extracted using 500 μL methanol and a tissue lyzer at 50 Hz for 30 s (3 times) followed with 5 ultrasonication cycles (1 min treatment and 1 min break). The supernatants were transferred to new tubes after centrifugation at 12000 rpm for 10 min at 4 °C, and followed by resuspension with a 15% methanol solution, followed by solid phase extraction (SPE) pretreated with MeOH and equilibrated with H_2O. Dried sample was resuspended in 100 μL methanol followed by filtration using a 0.22 μm membrane filter before UPLC -MS/MS analysis.

3) Liquid chromatography and mass spectrometry

UPLC-MS/MS analysis was conducted using an Agilent UPLC-MS/MS system consisting of an 1290 UPLC-system coupled with an Agilent 6470 triple-quadrupole mass spectrometer (Agilent Technologies, USA). For the analysis, 3 μL of the extraction was injected. Chromatographic separation was achieved on an Agilent ZORBAX RRHD Eclipse XDB C18 column (2.1 × 100 mm, 1.8 μm particles) using a flow rate of 0.65 mL/min at 45 °C during a 13 min gradient (0-12 min from 68% A to 20% A, 12-13 min 5%A), while using the solvents A, water containing 0.005% formic acid, and B, acetonitrile containing 0.005% formic acid. Electrospray ionization was performed in the negative ion mode using N2 at a pressure of 30 psi for the nebulizer with a flow of 10 L/min and a temperature of 300 °C, respectively. The sheath gas temperature was 350 °C with a flow rate of 11 L/min. The capillary was set at 3500 V and the nozzle voltage was 500 V. Multiple reaction monitoring (MRM) has been used for quantification of screening fragment ions.

4) Data preprocessing

Peak determination and peak area integration was performed using a MassHunter Workstation software (Agilent, Version B.08.00) while auto-integration was manually inspected and corrected if necessary. The obtained peak areas of targets were corrected by appropriate internal standards (IS) and calculated response ratios were used throughout the analysis.

Quantitative real-time PCR

Total RNA was extracted from human and mouse lungs with TRIzol regent (Invitrogen, Carlsbad, CA, USA) and the concentration of total RNA was quantified by the optical density at 260 nm according to the manufacturer's instructions. Then, complementary DNA (cDNA) was generated from 1 μg of total RNA using a cDNA reverse transcription kit (KR103, Tiangen Biotechnology, Beijing, China). The amplification and detection were performed using a Bio-Rad IQ5 system (Bio-Rad, Hercules, CA, USA). The SYBR Green I Q-PCR kit (TransGen Biotech, Beijing, China) was used for the amplification and the primer list is shown in the online table S8. Gene expression, the relative level of mRNA, was calculated using the $2^{-\Delta\Delta Ct}$ method [4] and normalized to β-actin mRNA expression level.

Western blotting

Total protein was isolated from human and mouse lungs using RIPA lysis buffer supplemented with protease and phosphatase inhibitor cocktail (P0013b, Beyotime, China). Protein concentration was determined by using a BCA Protein Assay Kit (Thermo Fisher Scientific, Waltham, MA, USA). Protein samples (20 μg) were analyzed through a gradient 8-12% sodium salt (SDS)-Polyacrylamide gel electrophoresis (PAGE) and transferred onto a nitrocellulose membrane according to manufacturer's instructions. The membranes were blocked for 1 h with 5% non-fat dry milk at room temperature and incubated overnight with the specific primary rabbit polyclonal antibodies at 4 °C, and then washed and probed with an appropriate anti-rabbit secondary antibody. The protein signal was visualized using a Tanon Automatic Chemiluminescence/Fluorescence Image Analysis System (5200, Tanon, China), and β-actin served as a loading control. ImageJ (the National Institutes of Health, Bethesda, MD, USA) was uesd to compare band intensity of respective targeted protein to a loading control. The antibodies used in the experiments are summarized in online table S9.

Histological staining and analysis

Counts of inflammatory cells in resuspended BALF were performed through a Giemsa staining. Inflammatory cells were counted using a IPWIN32 (Cybernetics Info Tech, Inc, America) depending on typical morphological characteristics, combined with the number of total cells and the collected BALF volume. Additionally, the percentage of macrophages, lymphocytes, and neutrophils was calculated.

The method of making mouse tissue sections (left lungs, livers, spleens, kidneys, and intestines) is the same as before we mentioned. HE staining to evaluate structures and chronic inflammation of tissues was performed. Chronic inflammation of lungs was evaluated using the method of Lv et al. [5], and the detail of the criteria was presented in the supplementary materials (see online table S10). Masson staining to analyzed the levels of collagen and evaluate the degree of pulmonary fibrosis was performed using the modified Ashcroft scale defined by Hübner et al. [6], which recommended the criteria showed in the supplementary materials (see online table S11).

Immunohistochemical (IHC) experiment was used to evaluate the immunoreactivity of Tbxas1, Hpgds, cPgds, and Ptges3 in paraffin-embedded human and mouse lung sections, and COL1 in paraffin-embedded mouse lung sections. Firstly, we used the rabbit anti-human and mouse antibody (see online table S12) to detect positive areas of human and mouse lung sections embedded in paraffin. Positive signals were visualized using goat anti-rabbit secondary antibody (see online table S12). Signal was developed using a 3-amino-9-ethylcarbazole reagent, and nuclei were stained using haematoxylin.

We performed Giemsa staining for each mouse in triplicate and pictures acquired from three random microscopic fields (at 20X magnification) for each image. We performed both HE and Masson staining for each mouse with three replicates and pictured no less than 30 microscopic random fields (at 20X magnification) for each image, and calculated the mean values of all evaluated images. IHC staining was performed for each mouse in triplicate and pictures acquired from six random microscopic fields (at 20X magnification) for each image. We analyzed all the above images by using IPWIN32 (Cybernetics Info Tech, Inc, America).

The analysis was performed independently by 2 operators. The antibodies used in the experiments are summarized in the supplementary materials (see online table S12).

Hydroxyproline assay

Concentrations of hydroxyproline in mouse lungs were measured using the Hydroxyproline Assay Kit (NBP2-59747, Novus biologicals, USA) according to the hydroxyproline assay protocol. The absorbance of all samples and standard curve wells were measured at 560 nm.

References

1. Uller L, Mathiesen JM, Alenmyr L, et al. Antagonism of the prostaglandin D2 receptor CRTH2 attenuates asthma pathology in mouse eosinophilic airway inflammation. Respir Res. 2007;8:16.
2. Suzuki Y, Inoue T, Yamamoto A, et al. Prophylactic effects of the histamine H1 receptor antagonist epinastine and the dual thromboxane A2 receptor and chemoattractant receptor homologous molecule expressed on Th2 cells antagonist Ramatroban on allergic rhinitis model in mice. Biol Pharm Bull. 2011;34:507-10.
3. Zhang S, Wu X, Yu S. Prostaglandin D2 receptor D-type prostanoid receptor 2 mediates eosinophil trafficking into the esophagus. Dis Esophagus. 2014;27:601-6.
4. Livak KJ, Schmittgen TD. Analysis of relative gene expression data using real-time quantitative PCR and the 2(-Delta Delta C(T)) Method. Methods. 2001;25(4):402-8.
5. Lv X, Wang X, Li K, et al. Rupatadine protects against pulmonary fibrosis by attenuating PAF-mediated senescence in rodents. PLoS ONE. 2013;8:e68631.
6. Hübner RH, Gitter W, El Mokhtari NE, et al. Standardized quantification of pulmonary fibrosis in histological samples. Biotechniques. 2008;44(4):507-11.

Figure S1

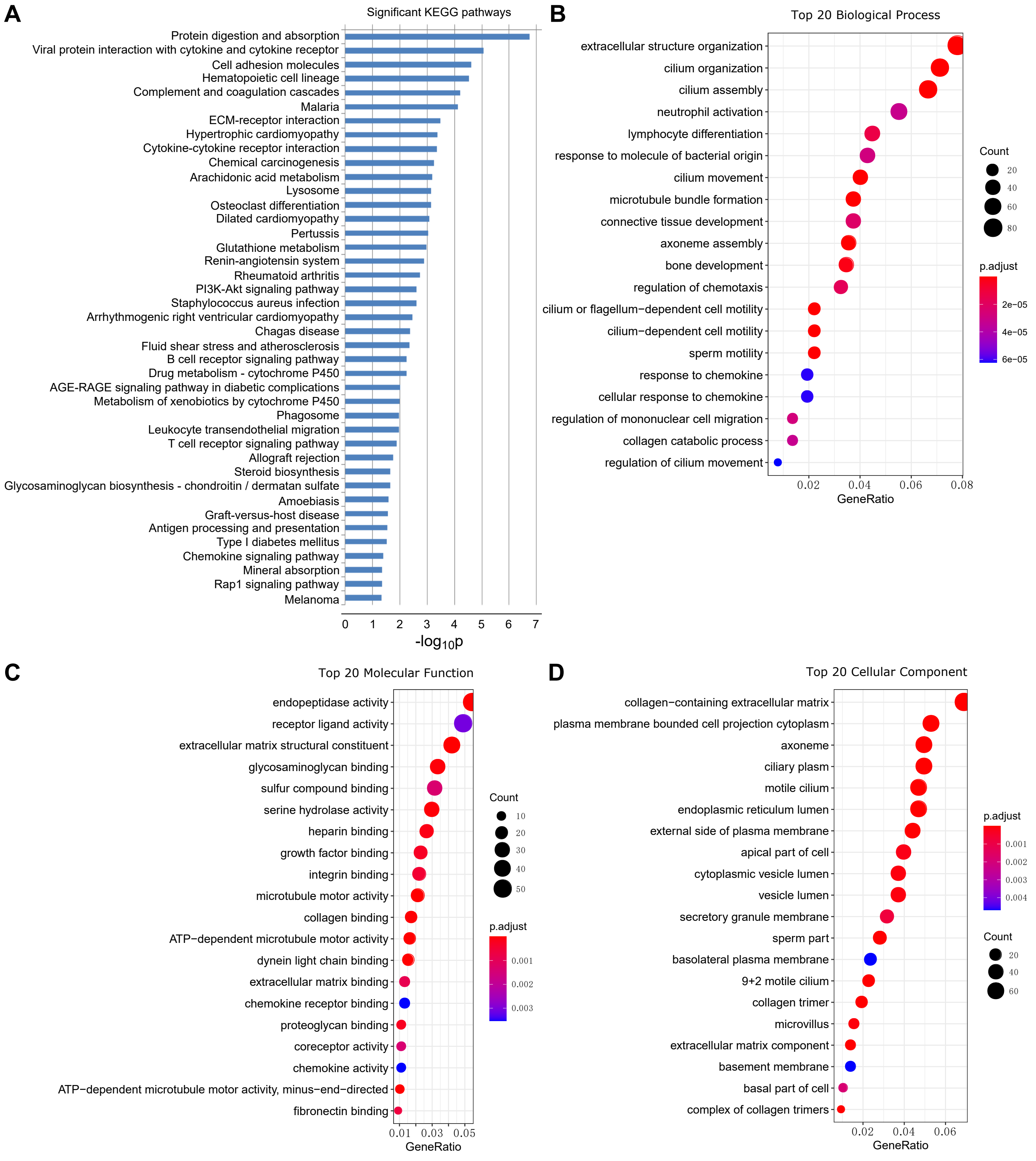
A
Significant KEGG pathways
Protein digestion and absorption
Viral protein interaction with cytokine and cytokine receptor
Cell adhesion molecules
Hematopoietic cell lineage
Complement and coagulation cascades
Malaria
ECM-receptor interaction
Hypertrophic cardiomyopathy
Cytokine-cytokine receptor interaction
Chemical carcinogenesis
Arachidonic acid metabolism
Lysosome
Osteoclast differentiation
Dilated cardiomyopathy
Pertussis
Glutathione metabolism
Renin-angiotensin system
Rheumatoid arthritis
PI3K-Akt signaling pathway
Staphylococcus aureus infection
Arrhythmogenic right ventricular cardiomyopathy
Chagas disease
Fluid shear stress and atherosclerosis
B cell receptor signaling pathway
Drug metabolism - cytochrome P450
AGE-RAGE signaling pathway in diabetic complications
Metabolism of xenobiotics by cytochrome P450
Phagosome
Leukocyte transendothelial migration
T cell receptor signaling pathway
Allograft rejection
Steroid biosynthesis
Glycosaminoglycan biosynthesis - chondroitin / dermatan sulfate
Amoebiasis
Graft-versus-host disease
Antigen processing and presentation
Type I diabetes mellitus
Chemokine signaling pathway
Mineral absorption
Rap1 signaling pathway
Melanoma
0 1 2 3 4 5 6 7
$-\log_{10}p$
B
Top 20 Biological Process
extracellular structure organization
cilium organization
cilium assembly
neutrophil activation
lymphocyte differentiation
response to molecule of bacterial origin
cilium movement
microtubule bundle formation
connective tissue development
axoneme assembly
bone development
regulation of chemotaxis
cilium or flagellum−dependent cell motility
cilium−dependent cell motility
sperm motility
response to chemokine
cellular response to chemokine
regulation of mononuclear cell migration
collagen catabolic process
regulation of cilium movement
0.02 0.04 0.06 0.08
GeneRatio
Count
20
40
60
80
p.adjust
2e−05
4e−05
6e−05
C
Top 20 Molecular Function
endopeptidase activity
receptor ligand activity
extracellular matrix structural constituent
glycosaminoglycan binding
sulfur compound binding
serine hydrolase activity
heparin binding
growth factor binding
integrin binding
microtubule motor activity
collagen binding
ATP−dependent microtubule motor activity
dynein light chain binding
extracellular matrix binding
chemokine receptor binding
proteoglycan binding
coreceptor activity
chemokine activity
ATP−dependent microtubule motor activity, minus−end−directed
fibronectin binding
0.01 0.03 0.05
GeneRatio
Count
10
20
30
40
50
p.adjust
0.001
0.002
0.003
D
Top 20 Cellular Component
collagen−containing extracellular matrix
plasma membrane bounded cell projection cytoplasm
axoneme
ciliary plasm
motile cilium
endoplasmic reticulum lumen
external side of plasma membrane
apical part of cell
cytoplasmic vesicle lumen
vesicle lumen
secretory granule membrane
sperm part
basolateral plasma membrane
9+2 motile cilium
collagen trimer
microvillus
extracellular matrix component
basement membrane
basal part of cell
complex of collagen trimers
0.02 0.04 0.06
GeneRatio
p.adjust
0.001
0.002
0.003
0.004
Count
20
40
60

Figure S2

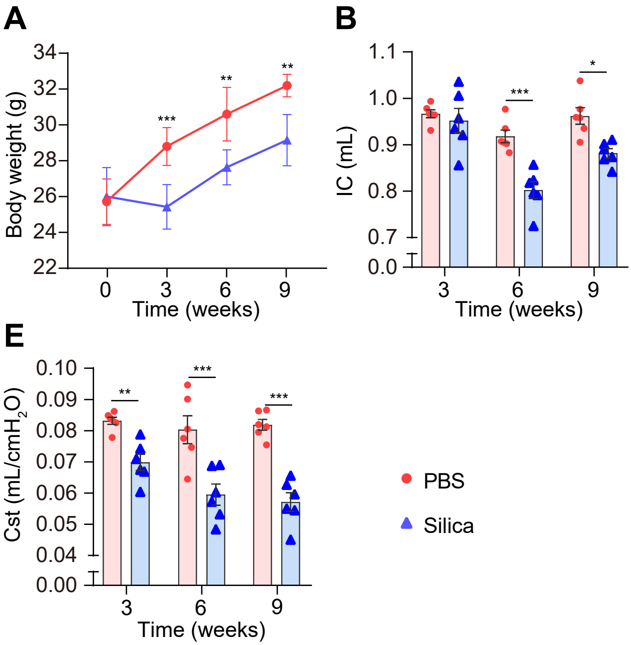
A
Body weight (g)
Time (weeks)
B
IC (mL)
Time (weeks)
C
Rrs (cmH2O·s/mL)
Time (weeks)
D
Crs (mL/cmH2O)
Time (weeks)
E
Cst (mL/cmH2O)
Time (weeks)
PBS
Silica

Figure S3

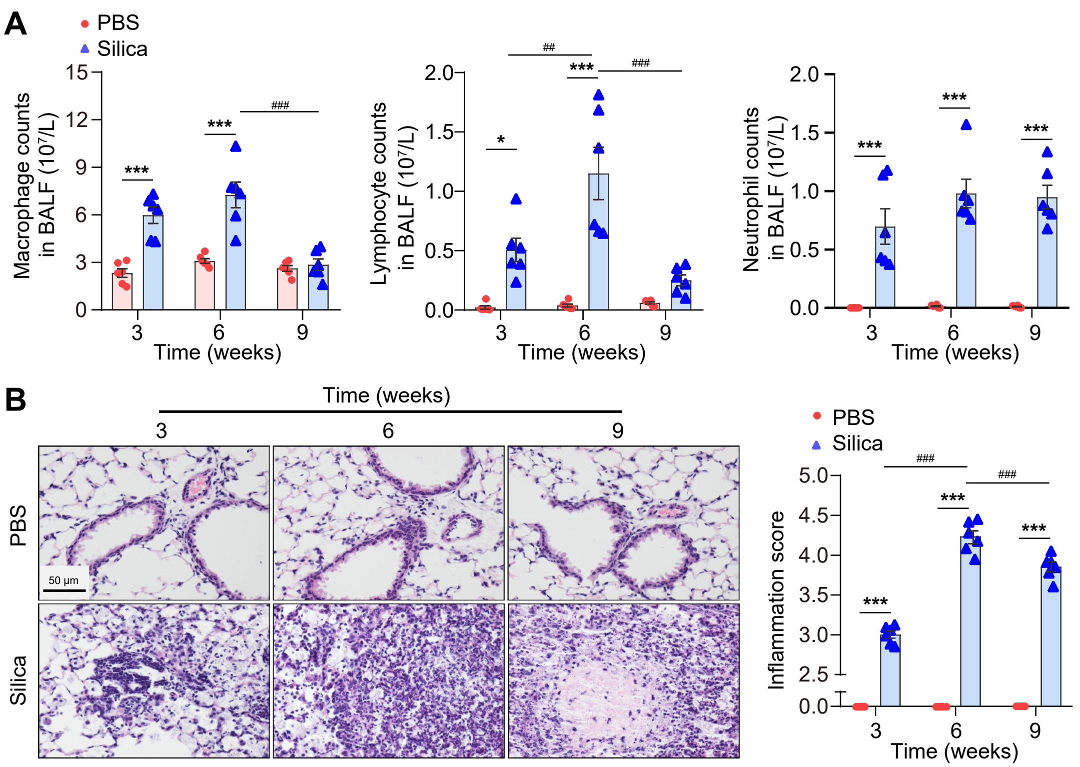

A
PBS
Silica
Macrophage counts in BALF (10^7/L)
Lymphocyte counts in BALF (10^7/L)
Neutrophil counts in BALF (10^7/L)
Time (weeks)
B
Time (weeks)
3
6
9
PBS
Silica
50 μm
Inflammation score

Figure S4

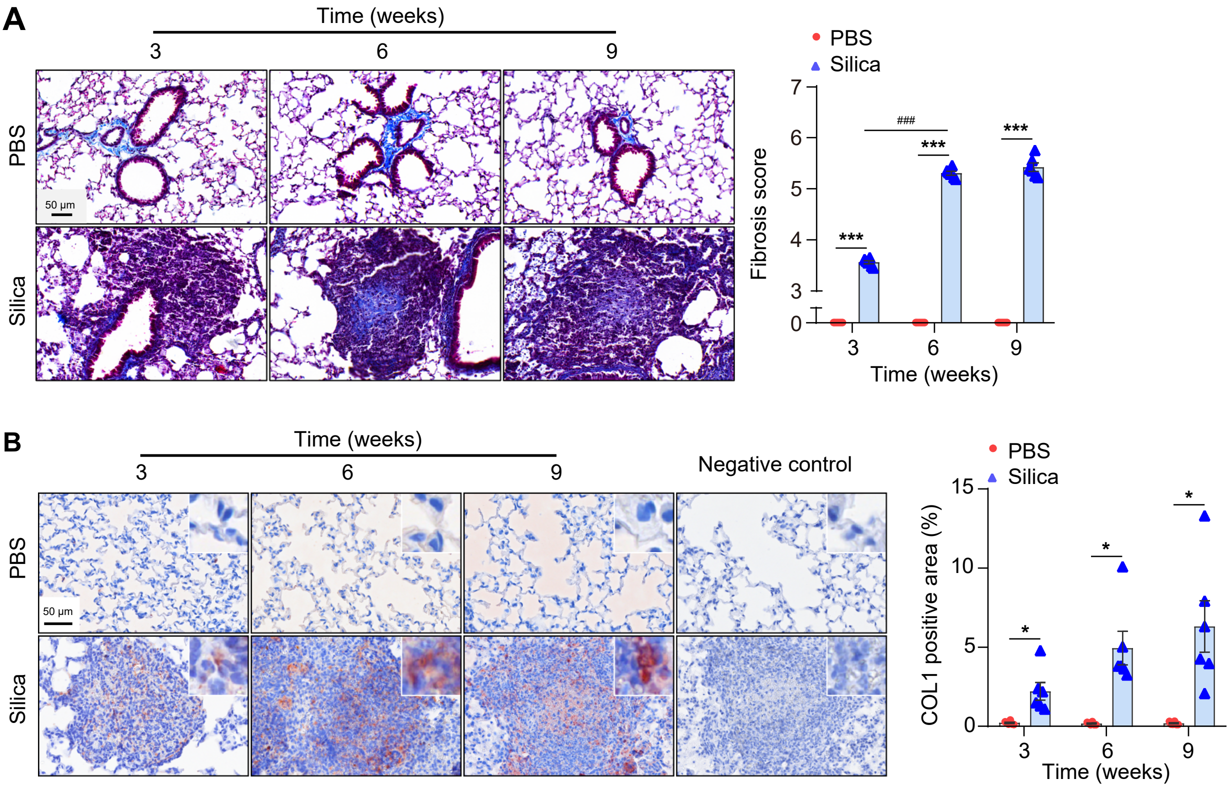

A
Time (weeks)
3
6
9
PBS
Silica
50 μm
PBS
Silica
Fibrosis score
7
6
5
4
3
0
***
###
***
***
Time (weeks)
B
Time (weeks)
Negative control
50 μm
PBS
Silica
COL1 positive area (%)
15
10
5
0
*
*
*
Time (weeks)

Figure S5

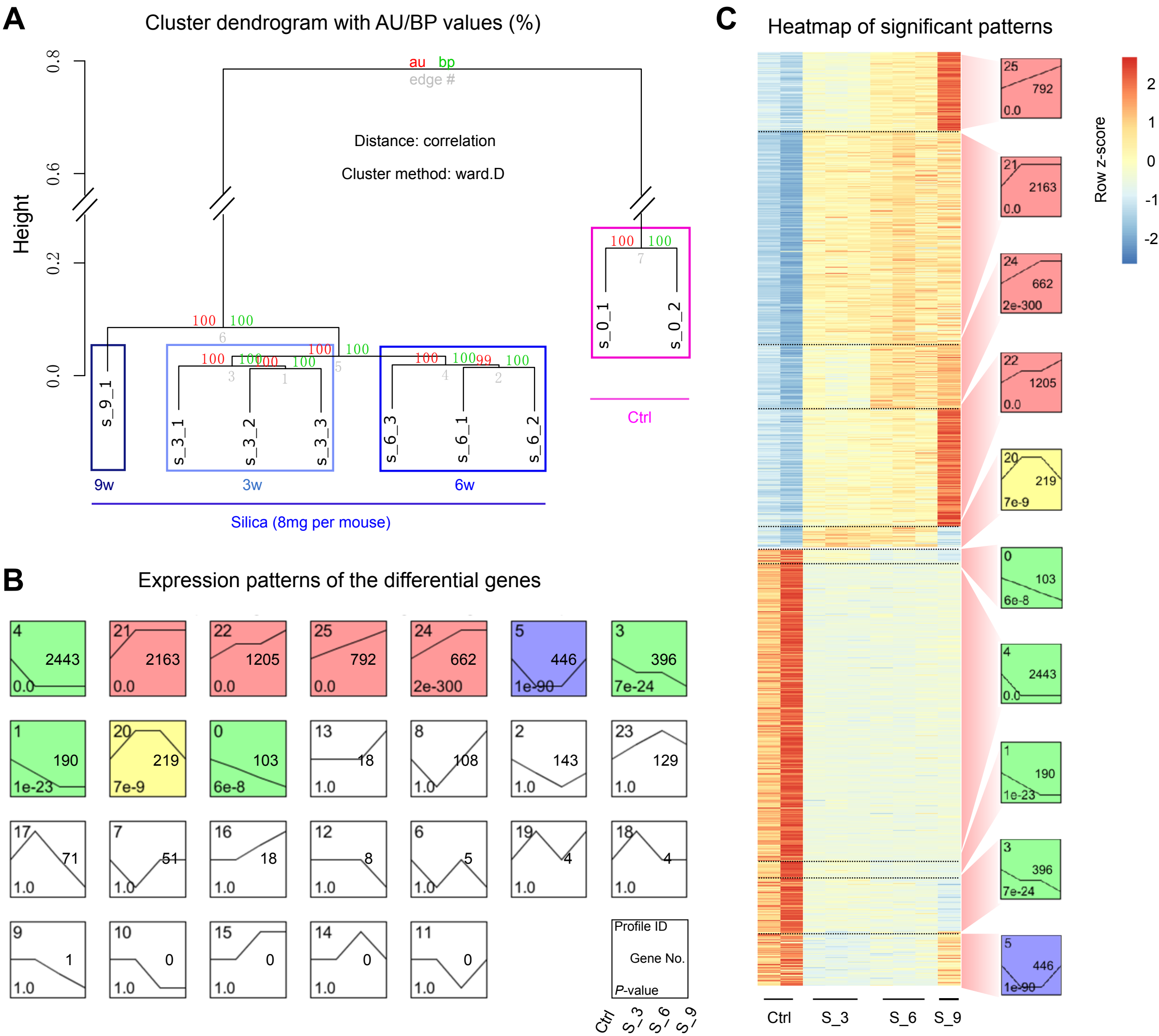
A
Cluster dendrogram with AU/BP values (%)
au bp
edge #
Distance: correlation
Cluster method: ward.D
Height
0.8
0.6
0.2
0.0
s_9_1
s_3_1
s_3_2
s_3_3
s_6_3
s_6_1
s_6_2
s_0_1
s_0_2
9w
3w
6w
Ctrl
Silica (8mg per mouse)
B
Expression patterns of the differential genes
Profile ID
Gene No.
P-value
Ctrl
S_3
S_6
S_9
C
Heatmap of significant patterns
Row z-score
Ctrl
S_3
S_6
S_9

Figure S6

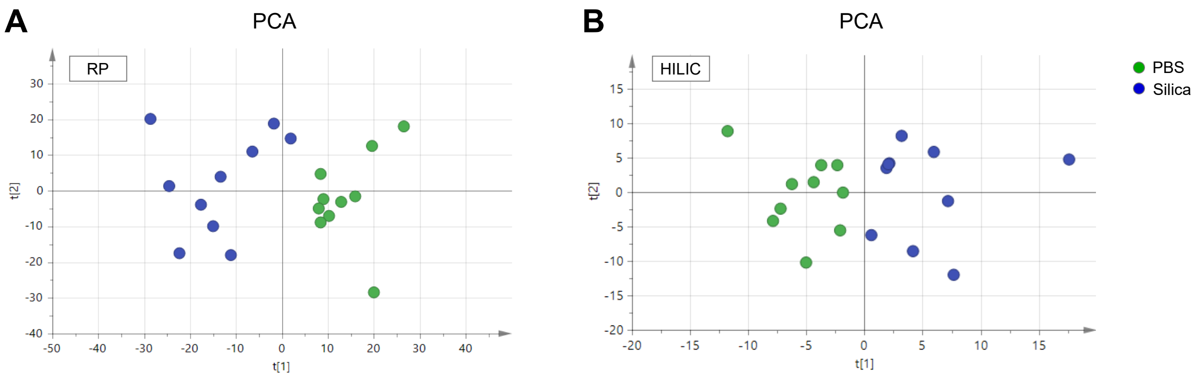
A
PCA
RP
t[2]
t[1]
30
20
10
0
-10
-20
-30
-40
-50
-40
-30
-20
-10
0
10
20
30
40
B
PCA
HILIC
t[2]
t[1]
15
10
5
0
-5
-10
-15
-20
-20
-15
-10
-5
0
5
10
15
PBS
Silica

Figure S7

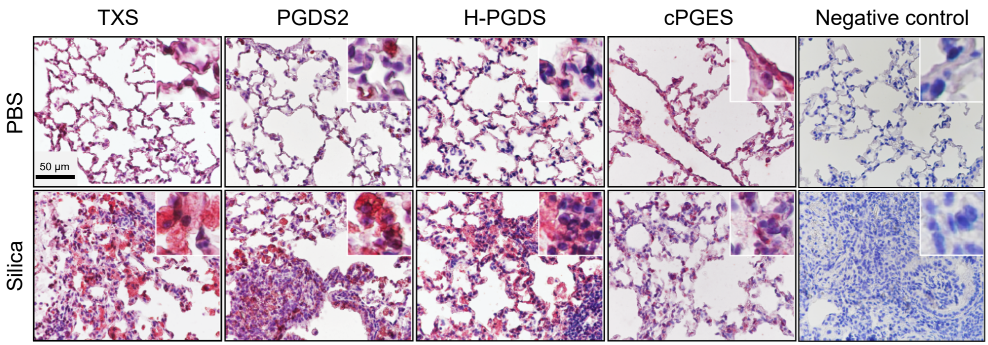

Figure S8

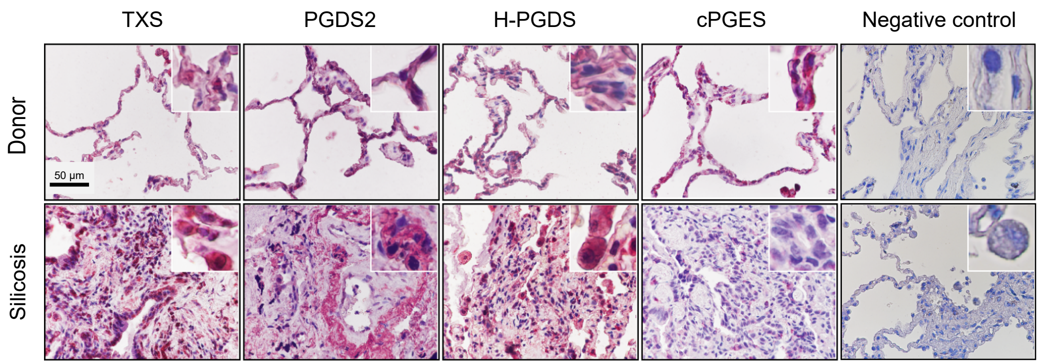

TXS
PGDS2
H-PGDS
cPGES
Negative control
Donor
Silicosis
50 μm

Figure S9

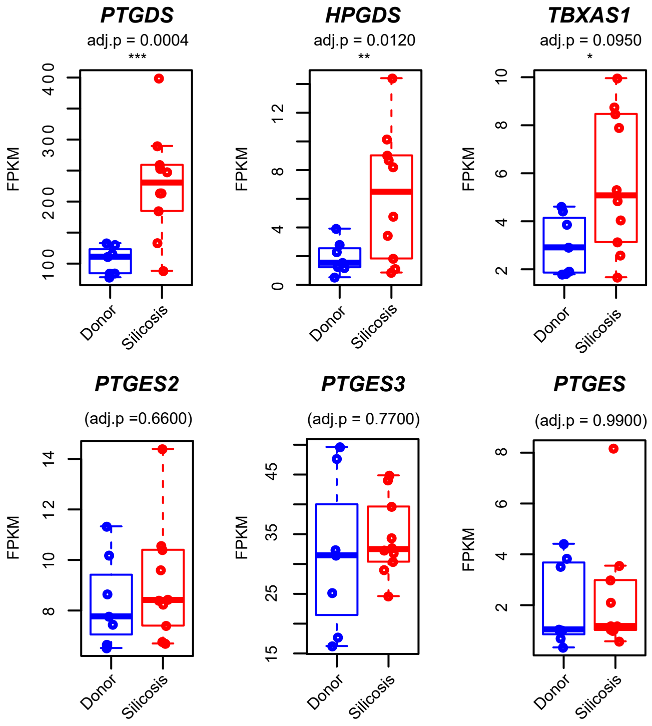

PTGDS
adj.p = 0.0004
***
HPGDS
adj.p = 0.0120
**
TBXAS1
adj.p = 0.0950
*
PTGES2
(adj.p =0.6600)
PTGES3
(adj.p = 0.7700)
PTGES
(adj.p = 0.9900)
FPKM
Donor
Silicosis

Figure S10

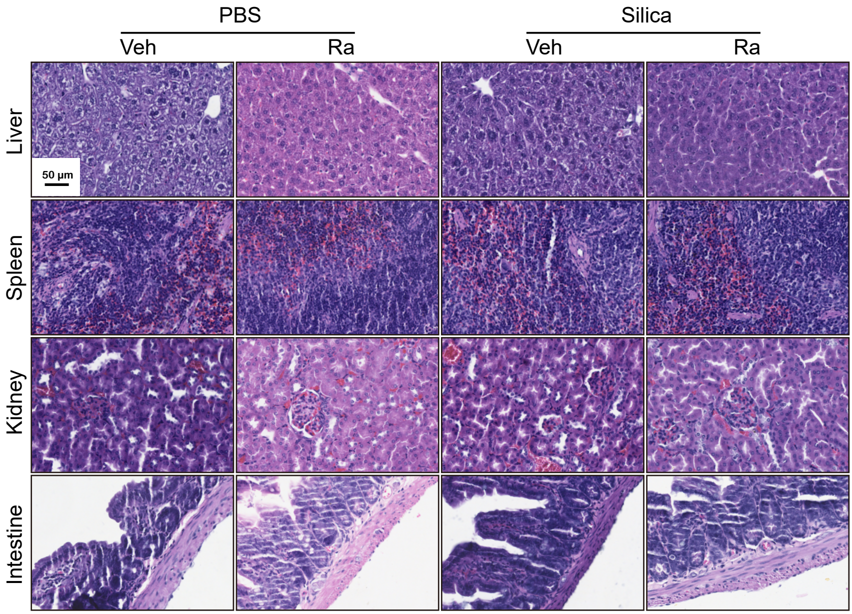

Figure S11

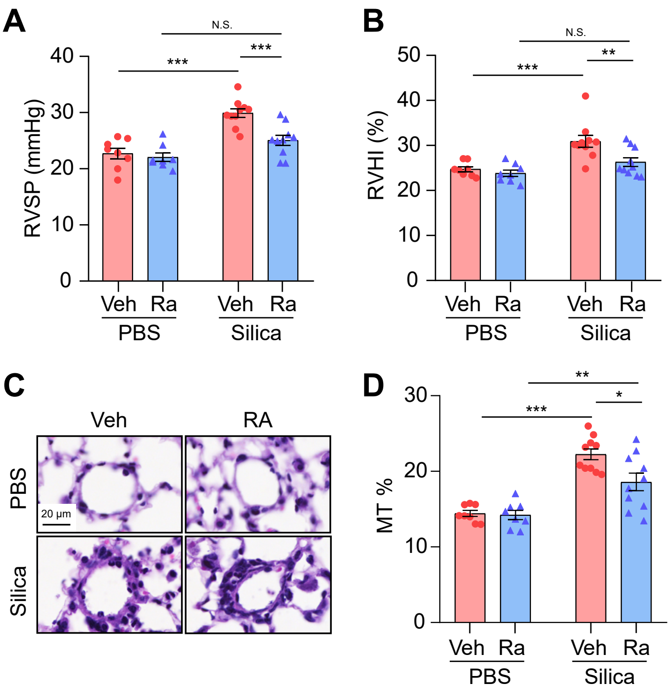
A
N.S.
***
***
RVSP (mmHg)
40
30
20
10
0
Veh
Ra
Veh
Ra
PBS
Silica
B
N.S.
**
***
RVHI (%)
50
40
30
20
10
0
Veh
Ra
Veh
Ra
PBS
Silica
C
Veh
RA
PBS
Silica
20 µm
D
**
*
***
MT %
30
20
10
0
Veh
Ra
Veh
Ra
PBS
Silica

Figure S12

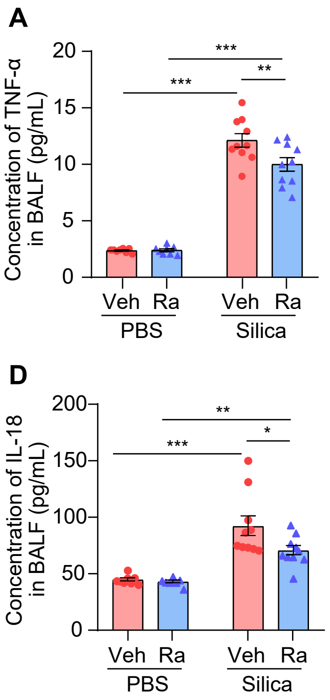
A
B
C
D
Concentration of TNF-α in BALF (pg/mL)
Concentration of IL-6 in BALF (pg/mL)
Concentration of IL-1β in BALF (pg/mL)
Concentration of IL-18 in BALF (pg/mL)
Veh
Ra
PBS
Silica
***
**
*

Figure S13

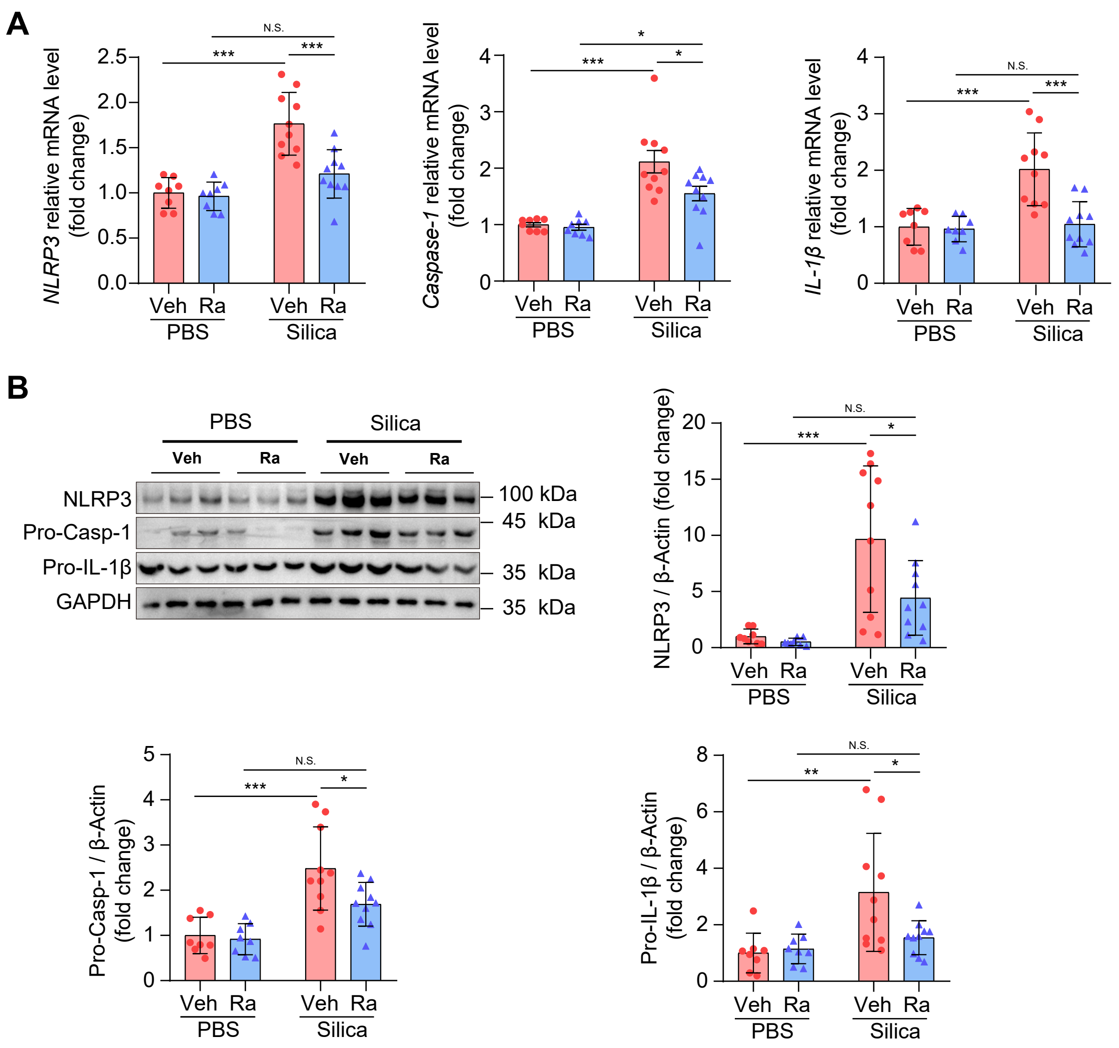
A
NLRP3 relative mRNA level (fold change)
Caspase-1 relative mRNA level (fold change)
IL-1β relative mRNA level (fold change)
N.S.
***
*
Veh Ra
PBS
Silica
B
PBS
Silica
Veh
Ra
NLRP3
Pro-Casp-1
Pro-IL-1β
GAPDH
100 kDa
45 kDa
35 kDa
35 kDa
NLRP3 / β-Actin (fold change)
Pro-Casp-1 / β-Actin (fold change)
Pro-IL-1β / β-Actin (fold change)
**

Figure S14

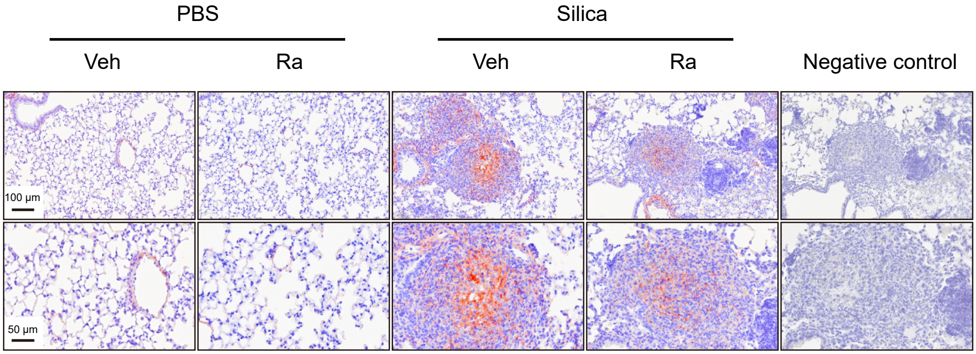

Supplementary figures

Figure S1

Functional enrichment analysis for the differentially altered genes in the lungs of silicosis patients. (A) The significant KEGG pathways. (B) The most significantly enriched biological processes. (C) The most significantly enriched cellular components. (D) The most significantly enriched items related to molecular functions. For B-D, the color indicates the significance of each item, while the dot size reflects the number of differential genes included in the corresponding items. KEGG, Kyoto Encyclopedia of Genes and Genomes; GO, gene ontology.

Figure S2

Body weight and pulmonary function of mice in the Silica group and the PBS group. (A) The body weight of mice in the Silica or PBS group, and the quantitative results are shown as mean ± SD. (B-E) Measurements of parameters of pulmonary function, including IC, Rrs, Crs, and Cst. Pulmonary function test was performed at least three times, and all the quantitative results are shown as mean ± SEM. The differences were analyzed by a two-way ANOVA and followed by Bonferroni adjustment. *: $p < 0.05$, **: $p < 0.01$, and ***: $p < 0.001$ are representative for the differences of the Silica group (n = 6 each group) as compared to the PBS group (n = 6 each group). PBS, phosphate-buffered saline; IC, inspiratory capacity; Rrs, resistance; Crs, compliance of the respiratory system; Cst, quasi-static lung compliance.

Figure S3

Changes of pulmonary inflammation in mice from the Silica group *versus* the PBS group at different time points (3 weeks, 6 weeks, and 9 weeks). (A) Counts of inflammatory cells (macrophages, lymphocytes, and neutrophils) in BALF. (B) Representative images of hematoxylin-eosin staining and quantification of pulmonary inflammation. All scale bars = 50 μm. All the above experiments were performed at least three times. Quantitative analysis results are presented as mean ± SEM. The differences were analyzed by a two-way ANOVA and followed by Bonferroni adjustment. ***: $p < 0.001$ are representative for the differences from the Silica group (n = 6) *versus* the PBS group (n = 6); $^{\#}$: $p < 0.05$, and $^{\#\#\#}$: $p < 0.001$ are representative for the differences from different time points after silica instillation. PBS, phosphate-buffered saline; BALF, bronchoalveolar lavage fluid.

Figure S4

Changes of pulmonary fibrosis in mice from the Silica group *versus* the PBS group at different time points (3 weeks, 6 weeks, and 9 weeks). (A) Representative images of Masson staining and quantification of pulmonary fibrosis. (B) Representative images of immunohistochemical staining and quantification of immunoreactivity of COL1 in mouse lung sections. Red-brown indicates the positive staining. All scale bars = 50 μm. All the above experiments were performed at least three times. Quantitative analysis results are presented as mean ± SEM. The differences were analyzed by a two-way ANOVA and followed by Bonferroni adjustment. **: $p < 0.01$ and ***: $p < 0.001$ are representative for

the differences from the Silica group (n = 6) *versus* the PBS group (n = 6); ###: p < 0.001 are representative for the differences from different time points after silica instillation. PBS, phosphate-buffered saline; COL, collagen.

Figure S5

The time-course transcriptional analysis of silica-induced mouse lungs. (A) Hierarchical clustering of the RNA sequencing samples. The y-axis represents the distance coefficient between samples. (B) All the possible gene expression patterns identified by the STEM method. The x-axis represents the time points during the model construction, and the following timelines were used for each group: Silica, 0 week (S_0), Silica, 3 weeks (S_3), Silica, 6 weeks (S_6) and Silica, 9 weeks (S_9). For each cluster, the cluster ID (top left corner), the p value (lower left corner), the number of genes contained in the cluster (right middle) are reported. The clusters with a colored background are those that are significant. (C) The heatmap of significant patterns. Each row represents a gene. The expression level of genes were normalized using row z-score. STEM, short time-series expression miner.

Figure S6

PCA of the samples performed using the LC-MS-based untargeted metabolomics. (A) PCA analysis using the compounds that were selected through the RP. (B) PCA analysis using the compounds selected with the HILIC. The green color represents the PBS group, while the blue color the silica group. PCA, principal component analysis; LC-MS, liquid chromatography-tandem mass spectrometry; RP, reversed-phase; HILIC, hydrophilic interaction liquid chromatography; PBS, phosphate-buffered saline.

Figure S7

Representative images and negative controls of immunohistochemical staining of TXS, PGDS2, H-PGDS, and cPGES in mouse lung sections from the Silica or PBS group. Red-brown indicates the positive staining. All scale bars = 50 μm. PBS, phosphate-buffered saline.

Figure S8

Representative images and negative controls of immunohistochemical staining of TXS, PGDS2, H-PGDS, and cPGES in human lung sections from silicosis patients or healthy donors. Red-brown indicates the positive staining. All scale bars = 50 μm.

Figure S9

Comparison of the expression levels for the genes encoding PGD_2 and TXA_2 synthases through the RNA sequencing in the lungs of healthy donors and silicosis patients. The differences between the two groups were analyzed by negative binomial GLM fitting and Wald statistics through DESeq2 package. *: adj.p < 0.1, **: adj.p < 0.05 and ***: adj.p < 0.001 are representative for the differences between the silicosis group (n = 10) as compared to the donor group (n = 7). Adj.p, adjusted p-value.

Figure S10

The representative images of hematoxylin-eosin staining of liver, spleen, kidney, and intestine in a silica-induced mouse model treated with Ramatroban. All scale bars = 50 μm. Veh, vehicle; Ra, ramatroban; PBS, phosphate-buffered saline.

Figure S11

The impact of Ramatroban treatment on right heart function and pulmonary artery medical thickness in a silica-induced mouse model. (A) The RVSP of mice. (B) The RVHI of mice. (C) Representative images of HE staining of pulmonary vascular in lung sections. (D) Quantification of the percentage of pulmonary artery media thickness (MT%). All the quantitative results are presented as mean ± SEM. The differences were analyzed by a two-way ANOVA and followed by Bonferroni adjustment. N.S.: no significance; *: $p < 0.05$, **: $p < 0.01$, and ***: $p < 0.001$. PBS group: n = 8 each group; Silica group: n = 10 each group. RVSP, right ventricular systolic pressure; RVHI, right ventricular hypertrophy index; Veh: vehicle; Ra: Ramatroban; PBS, phosphate-buffered saline.

Figure S12

The impact of Ramatroban treatment on proinflammatory cytokines in supernatants of BALF in a silica-induced mouse model. (A) Concentration of TNF-α in BALF. (B) Concentration of IL-6 in BALF. (C) Concentration of IL-1β in BALF. (D) Concentration of IL-18 in BALF. The experiment was performed at least three times. All the quantitative results are presented as mean ± SEM. The differences were analyzed by a two-way ANOVA and followed by Bonferroni adjustment. *: $p < 0.05$, **: $p < 0.01$, and ***: $p < 0.001$. PBS group: n = 8 each group; Silica group: n = 10 each group. BALF, bronchoalveolar lavage fluid; TNF-α, tumor necrosis factor-alpha; IL-6, interleukin-6; IL-1β, interleukin-1 beta; IL-18, interleukin-18; PBS, phosphate-buffered saline.

Figure S13

The impact of Ramatroban treatment on NLRP3 inflammasome in lung tissues in a silica-induced mouse model. (A) Relative mRNA level of *NLRP3*, *Caspase-1*, and *IL-1β.* (B) Representative western blotting images and quantification of proteins (NLRP3, pro-Casp-1, and pro-IL-1β). The experiment was performed at least three times. All the quantitative results are presented as mean ± SEM. The differences were analyzed by a two-way ANOVA and followed by Bonferroni adjustment. N.S.: no significance; *: $p < 0.05$, **: $p < 0.01$, and ***: $p < 0.001$. PBS group: n = 8 each group; Silica group: n = 10 each group. Veh: vehicle; Ra: Ramatroban; NLRP3: Nucleotide-binding domain like receptor protein 3; IL-1β, interleukin-1 beta; PBS, phosphate-buffered saline.

Figure S14

Representative images and negative controls of immunohistochemical staining of collagen I in lung sections of a silica-induced mouse model treated with Ramatroban. Red-brown color indicates the positive staining. Upper scale bar indicates 100 μm, while lower scale bar indicates 50 μm. Veh, vehicle; Ra, ramatroban.
